# Short Duration Alagebrium Chloride Therapy Prediabetes Does Not Inhibit Progression to Autoimmune Diabetes in an Experimental Model

**DOI:** 10.3390/metabo11070426

**Published:** 2021-06-28

**Authors:** Danielle J. Borg, Pouya Faridi, Kai Lin Giam, Peta Reeves, Amelia K. Fotheringham, Domenica A. McCarthy, Sherman Leung, Micheal S. Ward, Brooke E. Harcourt, Rochelle Ayala, Jean L. Scheijen, David Briskey, Nadine L. Dudek, Casper G. Schalkwijk, Raymond Steptoe, Anthony W. Purcell, Josephine M. Forbes

**Affiliations:** 1 Glycation and Diabetes Complications, Mater Research Institute, The University of Queensland, Translational Research Institute, Brisbane, QLD 4102, Australia; danielle.borg@mater.uq.edu.au (D.J.B.); amelia.fotheringham@mater.uq.edu.au (A.K.F.); domenica.mccarthy@mater.uq.edu.au (D.A.M.); sherman.leung@mater.uq.edu.au (S.L.); michael.ward5@msd.com (M.S.W.); brooke.harcourt@unimelb.edu.au (B.E.H.); 2 Pregnancy and Development, Mater Research Institute, The University of Queensland, South Brisbane, QLD 4101, Australia; 3 Infection and Immunity Program, Department of Biochemistry and Molecular Biology, Biomedicine Discovery Institute, Monash University, Melbourne, VIC 3800, Australia; pouya.faridi@monash.edu (P.F.); kailin.giam@thermofisher.com (K.L.G.); rochelle.ayala.perez@monash.edu (R.A.); nadinedudekart@gmail.com (N.L.D.); anthony.purcell@monash.edu (A.W.P.); 4 Tolerance and Autoimmunity Group, The University of Queensland Diamantina Institute, Translational Research Institute, Brisbane, QLD 4102, Australia; peta.zorzetto@gmail.com (P.R.); r.steptoe@uq.edu.au (R.S.); 5 Murdoch Children’s Research Institute, Royal Children’s Hospital, Melbourne, VIC 3052, Australia; 6 Laboratory for Metabolism and Vascular Medicine, Department of Internal Medicine, Maastricht University, 6211 Maastricht, The Netherlands; j.scheijen@maastrichtuniversity.nl (J.L.S.); c.schalkwijk@maastrichtuniversity.nl (C.G.S.); 7 Cardiovascular Research Institute Maastricht, 6211 Maastricht, The Netherlands; 8 School of Human Movement and Nutrition Sciences, The University of Queensland, Brisbane, QLD 4067, Australia; d.briskey@uq.edu.au; 9 Mater Clinical School, The University of Queensland, Brisbane, QLD 4101, Australia

**Keywords:** advanced glycation end products, alagebrium chloride, cross-link breaker, immunopeptidome, MIN6N8 cell line, NOD mouse, autoimmune diabetes, type 1 diabetes

## Abstract

Mechanisms by which advanced glycation end products (AGEs) contribute to type 1 diabetes (T1D) pathogenesis are poorly understood. Since life-long pharmacotherapy with alagebrium chloride (ALT) slows progression to experimental T1D, we hypothesized that acute ALT therapy delivered prediabetes, may be effective. However, in female, non-obese diabetic (NOD*ShiLt*) mice, ALT administered prediabetes (day 50–100) did not protect against experimental T1D. ALT did not decrease circulating AGEs or their precursors. Despite this, pancreatic β-cell function was improved, and insulitis and pancreatic CD45.1^+^ cell infiltration was reduced. Lymphoid tissues were unaffected. ALT pre-treatment, prior to transfer of primed GC98 CD8^+^ T cell receptor transgenic T cells, reduced blood glucose concentrations and delayed diabetes, suggesting islet effects rather than immune modulation by ALT. Indeed, ALT did not reduce interferon-γ production by leukocytes from ovalbumin-pre-immunised NOD*ShiLt* mice and NOD*scid* recipients given diabetogenic ALT treated NOD splenocytes were not protected against T1D. To elucidate β-cell effects, NOD-derived MIN6N8 β-cell major histocompatibility complex (MHC) Class Ia surface antigens were examined using immunopeptidomics. Overall, no major changes in the immunopeptidome were observed during the various treatments with all peptides exhibiting allele specific consensus binding motifs. As expected, longer MHC Class Ia peptides were captured bound to H-2D^b^ than H-2K^b^ under all conditions. Moreover, more 10–12 mer peptides were isolated from H-2D^b^ after AGE modified bovine serum albumin (AGE-BSA) treatment, compared with bovine serum albumin (BSA) or AGE-BSA+ALT treatment. Proteomics of MIN6N8 cells showed enrichment of processes associated with catabolism, the immune system, cell cycling and presynaptic endocytosis with AGE-BSA compared with BSA treatments. These data show that short-term ALT intervention, given prediabetes, does not arrest experimental T1D but transiently impacts β-cell function.

## 1. Introduction

Advanced glycation end products (AGEs) are formed by irreversible modifications of proteins or lipids with reducing sugars [1]. AGEs are synthesized endogenously in vivo and are ingested excessively in the western diet [2], although the availability of dietary AGEs and how they contribute to pathophysiological changes in the body remains to be fully understood [3]. AGEs are postulated to be regulated by multiple scavenger receptors expressed on a number of cell types, including the pattern recognition receptor for AGEs (RAGE), which can trigger events such as cellular adhesion and spreading [4,5], migration [6,7,8], and cytokine production [9] via nuclear factor kappa-light-chain-enhancer of activated B cells (NF-κB) activation [3].

Circulating AGEs are independent predictors of progression to type 1 diabetes (T1D) in islet cell autoantibody-positive children [10]. A smaller study in children and adolescents with pancreatic islet autoantibodies who were first-degree relatives of individuals with T1D, showed that progression to overt diabetes correlated with high circulating concentrations of AGEs [11]. In a population based nested case-control study, polymorphisms in the gene that encode for RAGE, advanced glycosylation end product-specific receptor (*AGER*), conferred increased T1D risk and reduced circulating soluble RAGE concentrations in young children with ketoacidosis carrying the HLA-DR3/DQ4 haplotype [12]. Additional studies have confirmed that in vitro excess AGE exposure results in pancreatic β-cell dysfunction reducing glucose stimulated insulin synthesis and secretion [11,13,14,15], increasing apoptosis [11,13], and mitochondrial abnormalities [11,16]. In vivo, acute AGE injections provided to healthy rodents [11,13], as well as chronic dietary AGE intake [17,18,19], each initiated β-cell dysfunction, resulting in insulin secretory deficiencies compared to control groups [11,13,16,17,18,19]. Temporal changes in circulating AGEs and RAGE expression are apparent pre-diabetes in the mouse model for T1D, non-obese diabetic (NOD) mice [20] when compared to non-diabetic NOD*scid* mice [21].

Thus, it is not surprising that anti-AGE therapies, such as diets low in AGEs [18], or prophylactic injections of soluble RAGE, a decoy receptor for cellular RAGE, reverse insulin secretion defects, dampen pancreatic islet immune cell infiltration and delay experimental autoimmune diabetes in NOD mice [22]. We have previously demonstrated that continuous, life-long administration of the therapeutic agent alagebrium (4,5-dimethyl-3-(2-oxo2-phenylethyl)-thiazolium chloride (ALT)), thought to cleave the intermediate α-dicarbonyls compounds which form AGEs [23,24], reduced diabetes progression in the NOD*ShiLt* mouse and protected β-cell function [11].

What is currently unknown is whether effects seen with AGE-lowering strategies, such as ALT on pancreatic β-cell function, are independent of improvements in the immune system in autoimmune diabetes. Secondly, it is unclear if a shorter duration of therapy prediabetes could delay autoimmune diabetes onset. To address this, we used the NOD mouse-derived β-cell MIN6N8 cell line and NOD strains with varying susceptibility to autoimmune diabetes and examined the efficacy of short-term ALT administration prior to diabetes onset, from day 50–100 of life on diabetes development.

## 2. Results

### 2.1. Short-Term AGE Lowering Therapy with ALT Prediabetes Does Not Impact Experimental Autoimmune Diabetes Development in NODShiLt Mice

**Figure 1:**
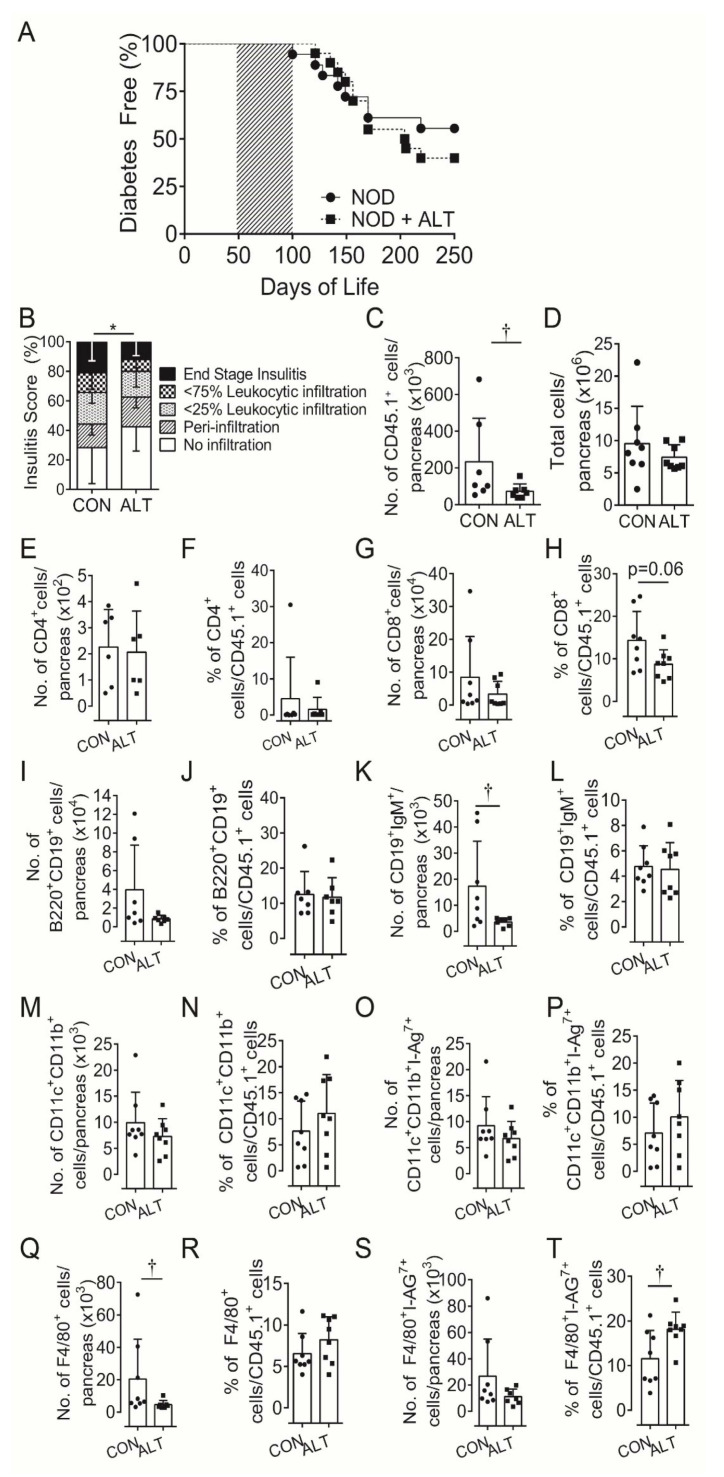
Short term AGE lowering therapy pre-diabetes in NOD mice does not protect against diabetes development despite attenuating β-cell infiltration. (**A**) Diabetes incidence of female NOD*ShiLt* (NOD) mice (*n* = 18–20/group) from day 50 to 100 of life (shaded grey) were untreated (NOD, ●) or treated with the AGE lowering therapy alagebrium chloride (ALT, 1 mg/kg/day, s.c; ■). Mice were monitored daily, blood glucose tested weekly for diabetes incidence and mice were euthanised when either blood glucose concentrations exceeded 15 mmol/L for two consecutive days, day 250 of life was reached without diabetes diagnosis. (**B**) Islet immune cell infiltration at day 90 of life shown as mean percentage of islets with no infiltrate (white), perivascular/periductal infiltrate (striped), <25% infiltrate (arrowhead), <75% infiltrate (checkerboard) or end stage insulitis (black) after no therapy (CON) or 40 days alagebrium chloride (ALT) treatment (*n* = 4 mice/group, 5 µm sections). After 30 days of ALT therapy, pancreata were digested and infiltrating immune cells quantified via surface staining for (**C**) total number of leukocyte common antigen positive (CD45.1^+^) immune cells, (**D**) total number and proportions of (**E**,**F**) CD4^+^ T cells, (**G**,**H**) CD8^+^ T cells, (**I**,**J**) CD19^+^B220^+^, (**K**,**L**) CD19^+^IgM^+^ B cells, (**M**,**N**) CD11b^+^CD11c^+^, (**O**,**P**) CD11b^+^CD11c^+^I-Ag7^+^, (**Q**,**R**) F4/80^+^ and (**S**,**T**) F4/80^+^I-Ag7^+^ immune cells (*n* = 7–8 mice/group; *n* = 2 experiments). Data represented as mean ± SD. For analysis in (**C**,**D**) data was log transformed. * *p* = 0.0005 vs. NOD (Chi-square); † *p* < 0.05 vs. NOD (Student’s *T* Test).

Previous studies have shown benefits of AGE-lowering therapies in arresting autoimmune diabetes development in NOD*ShiLt* mice [11]. Fifty days of daily ALT therapy from 50 to 100 days of life modestly delayed the age of diabetes onset (control 141 ± 32 days vs. ALT 171 ± 38 days, *p* = 0.05; Figure 1A), but there were no differences in disease incidence when compared to control mice by the study end (control: 11/18; alagebrium: 15/20 mice, *p* = 0.5; Figure 1A).

### 2.2. Short-Term Therapy with ALT Increases Insulin Secretion Pre-Diabetes

Beta cell function was examined in mice during and directly after ALT therapy. At approximately day 75 of life (after ~25 days of ALT therapy), intraperitoneal (IPGTT; Table 1; Figure S1A,B) and oral (OGTT; Table 1; Figure S1C,D) glucose tolerance tests were performed in two separate cohorts of mice. In both tests, insulin secretion across the test duration (AUC_INSULIN_) was significantly greater in mice given ALT therapy (Table 1; Figure S1A–D). In addition, the first phase insulin response and action during the first 15 min of the IPGTT was higher in ALT treated mice (Table 1, AUC_INSULIN_:AUC_GLUCOSE_; Figure S1B). By day 107 of life, just after the completion of the ALT treatment period, circulating non-fasted insulin concentrations appeared higher in ALT treated mice when compared to controls but did not reach significance (*p* = 0.06; Table 2). During OGTT but not IPGTT, AUC_GLUCOSE_ was greater in mice which were administered ALT (Table 1; Figure S1C).

Despite the slight elevation in circulating insulin concentrations over the course of ALT therapy (Table 2), blood glucose concentrations, body weight and glycated haemoglobin remained unchanged over the course of treatment (Table 2). When examined towards the end of the ALT treatment period (~40 days), pancreatic islets were a similar size to those seen in control mice (Figure S1E) and did not differ in insulin (Figure S1F,G) or proinsulin (Figure S3H,I) staining as compared to controls. Overall, these data imply that ALT may have a temporal effect on pancreatic islet function early in diabetes progression.

### 2.3. AGEs and Precursor Molecules Were Unaffected by Short Term ALT Therapy

AGE precursor molecules, the dicarbonyls methylglyoxal (MGO), glyoxal (GO), and 3-deoxyglucosone (3-DG) remained unchanged across the treatment period in NOD*ShiLt* mice receiving ALT therapy (Figure S2A–C). Although these dicarbonyls did not associate with changes in circulating insulin (data not shown), GO and 3-DG associated positively with glycated haemoglobin after 30 days of therapy (Figure S2D,E). Collectively, this data suggests that short-term, intensive alagebrium therapy does not reduce reactive dicarbonyl compounds or stop diabetes progression. There was a trend towards the AGE carboxyethyl-lysine (CEL) being reduced after ALT therapy, which was not observed with other AGE modifications such as carboxylmethyl-lysine (CML) and methylglyoxal-derived hydroimiadazolone (MG-H1) (Figure S2F).

### 2.4. Localised Pancreatic but Not Lymph Node or Splenic Immune Cells Are Modulated by Short-Term ALT Therapy

Based on the temporal effects on insulin secretion in the present study and our previous finding that long-term ALT administration reduced diabetes incidence in NOD mice [11], we hypothesized that local immunopathology within the pancreas may be altered. Semi-quantitative insulitis scoring of immune cell infiltrate after 40 days of ALT therapy confirmed reduced islet infiltration compared with control mice (Figure 1B). Whole pancreatic digests after 30 days of therapy showed a consistently lower number of CD45.1^+^ leukocytes per pancreas in ALT-treated mice as compared with untreated mice (Figure 1C). Total pancreatic cell numbers (Figure 1D) [25], did not differ between groups.

Within CD45.1^+^ cell populations in the pancreas (Figure S3A; gating strategy) the number and proportions of CD4^+^ T cells (Figure 1E,F), B220^+^CD19^+^B cells (Figure 1I,J), non-activated CD11b^+^CD11c^+^ (Figure 1M,N) and activated CD11b^+^CD11c^+^ I-Ag7^+^ conventional dendritic cells (cDC; Figure 1O,P) were similar between groups. However, the pancreatic CD45.1^+^ cell populations tended to contain a lower percentage of CD8^+^ T cells although numbers remained unchanged (Figure 1G,H) with ALT. There were fewer mature CD19^+^IgM^+^ B cells and F4/80^+^ macrophages numbers (Figure 1K,Q) but not proportions and (Figure 1L,R), and higher proportions but not numbers of activated F4/80^+^I-Ag7^+^ macrophages (Figure 1S,T) after ALT treatment compared to control.

Since islet antigen-specific T cells are commonly primed in the pancreatic lymph nodes by antigen-presenting cells [26,27], we next set out to verify whether the decreases in infiltrating pancreatic leukocytes with ALT were due to modulation of the local lymph node milieu. Flow cytometry analysis (Figure S3B; gating strategy), of pancreatic lymph node (pLN) cell suspensions isolated after 30–40 days of ALT therapy showed no change in the absolute numbers and proportions of CD4^+^ (Figure S4A,B) and CD8^+^ T cells (Figure S4I,J). The number and proportions of naïve (CD62^+^CD44^−^), effector/effector memory (CD62^−^CD44^+^) or central memory (CD62^+^CD44^+^) CD4^+^ (Figure S4C–H) or CD8^+^ (Supplementary Figure S4K–P) T cell subsets remained unchanged. Similarly, regulatory T cells (Treg; CD4^+^CD25^+^Foxp3^+^), thought to play a key role in autoimmune diabetes in NOD mice [28] and T1D in humans [29], were not different between groups (Figure S4Q–R). Similar to the pancreas, CD11b^+^CD11c^+^ cDC (Figure S5A,B), and CD11b^−^CD11c^+^ plasmacytoid dendritic cells (Figure S5C,D) remained unchanged between groups. Unlike the pancreas, CD19^+^B220^+^ B cells (Figure S4S,T) remained unchanged.

Flow cytometry analysis (Figure S3C; gating strategy) of cell suspensions prepared from spleens showed no change in numbers and proportions of dendritic cells (Figure S5E–H), CD4^+^ (Figure S6A–G), CD8^+^ (Figure S6I–P) T cells, Tregs (Figure S6Q,R) or B cells (Figure S6S,T) after ALT therapy, with the exception of a reduced proportion of central memory CD4^+^ T cells (Figure S6H). Collectively, these results suggest that mice exposed to short-term ALT therapy have reduced proportions of pancreatic CD8^+^ and splenic central memory CD4^+^ T cells that could be subsequent to changes in pancreatic antigen presenting cell proportions.

### 2.5. Pre-Treatment with ALT Delays Diabetes Development Following Adoptive Transfer of G9C8 CD8 T Cell Receptor (TCR) Transgenic Cytotoxic Lymphocytes

**Figure 2:**
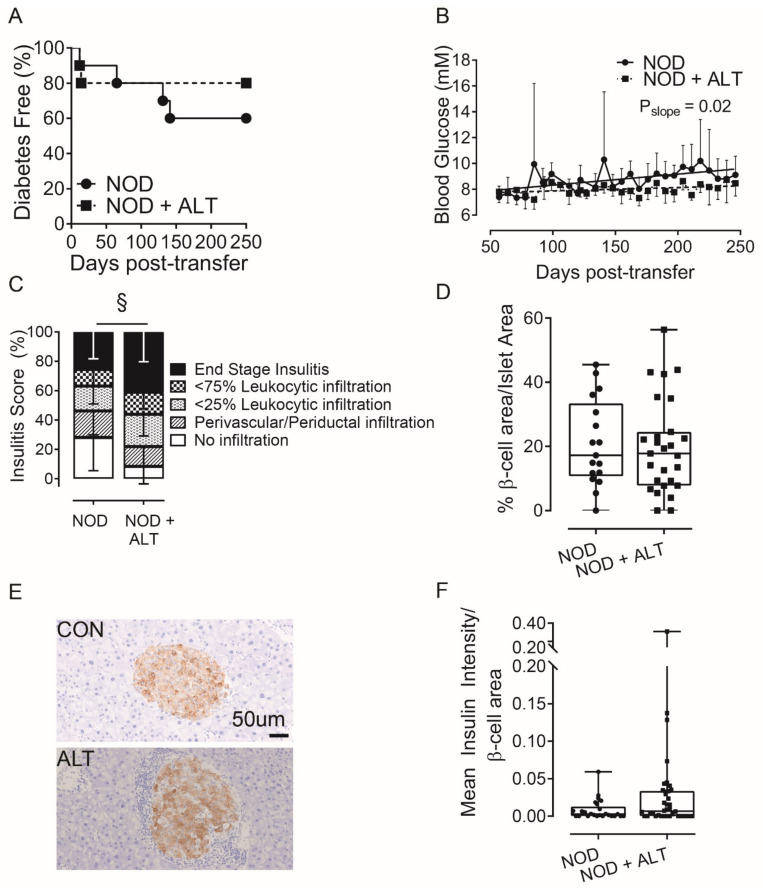
Alagebrium chloride delays diabetes development following adoptive transfer of G9C8 CD8 TCR transgenic cytotoxic lymphocytes. Diabetogenic splenocytes from NOD G9C8 CD8 TCR transgenic donors pre-primed with the aa15–23 of the insulin B chain (1 × 10^7^ cells), were transferred into non-diabetic NOD*ShiLt* recipients (*n* = 10/group; day 80 of life). NOD*ShiLt* recipients were pre-treated for 30 days with (NOD + ALT, 1 mg/kg/day, s.c; ■, dashed line) or without alagebrium chloride (NOD, ●, solid line). The recipient mice were monitored daily, tested for blood glucose and diabetes diagnosed by two consecutive blood glucose measurements > 15 m·mol/L and followed until diabetes diagnosis or until study end (day 250). (**A**) Diabetes incidence in NOD*ShiLt* recipients post-transfer of G9C8 cells. (**B**) Linear regression of non-fasting blood glucose concentrations over time, difference of slopes *p* = 0.02. (**C**) Pancreatic immune cell infiltration at day 250 (*n* = 6–8 mice/group), shown as mean percentage of islets with no infiltrate (white), perivascular/periductal infiltrate (striped), <25% infiltrate (arrowhead), <75% infiltrate (checkerboard) or end stage insulitis (black) of untreated splenocytes (CON; circles) or Alagebrium-treated splenocytes (ALT; squares) § *p* <0.0001, chi-square test. (**D**) β-cell area, determined from insulin IHC staining in (**E**) Representative images of islets detected with anti-insulin antibody in pancreatic sections taken from untreated (CON) or treated mice (ALT), scale bar = 50 µm. (**F**) Pancreatic expression of insulin (*n* = 26–40 sections/group, *n* = 2–39 islets/section, *n* = 6–8 mice/group, 5 µm sections, 96 µm apart. Data reported as either mean ± SD or box and whisker plots reporting median, interquartile ranges and min and max values.

To further explore the effects of ALT on β-cells during diabetes development, we adoptively transferred activated, diabetogenic NOD G9C8 CD8 TCR transgenic cytotoxic T lymphocytes, that recognize aa15–23 of the insulin B chain [30], into non-diabetic NOD*ShiLt* recipient mice that were pre-treated for 30-days with or without ALT. ALT delayed diabetes onset by an average of 18 days compared to controls and halved the number of mice which developed diabetes (20% incidence with ALT pre-treatment and 40% incidence in untreated mice; Figure 2A). ALT pre-treatment decreased non-fasting blood glucose concentrations across the study duration (*p* = 0.02, Figure 2B). However, ALT pre-treatment exacerbated insulitis (Figure 2C), although this did not overtly reduce β-cell area (Figure 2D). Pancreatic expression of insulin (Figure 2E,F) remained unchanged.

### 2.6. ALT Maintains Systemic Immune Function in the NODShiLt Mouse

**Figure 3:**
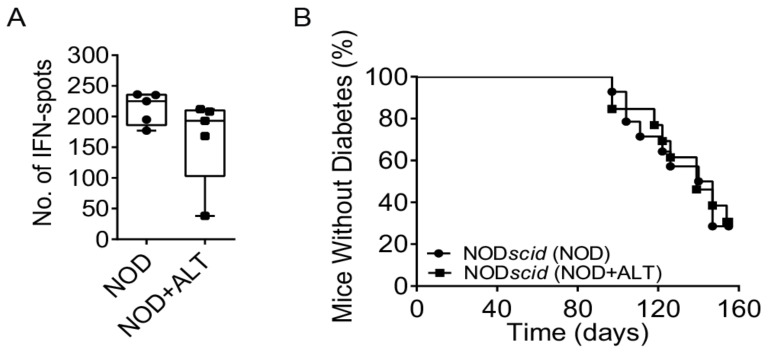
Systemic immune cells retain their function following short-term AGE lowering therapy with alagebrium chloride (ALT). (**A**) NOD*ShiLt* control mice (NOD, ●) and NOD*ShiLt* treated with alagebrium (NOD + ALT, 1 mg/kg/day, s.c; ■) were immunised with OVA/QuilA and 1 week later spleens harvested and the number of cells producing IFN-γ in responses to OVA restimulation enumerated with ELISpot. (*n* = 3 replicates from *n* = 5 mice/group, *n* = 2 independent experiments). (**B**) Diabetes incidence after adoptive transfer of control or ALT treated (for ~30 days) diabetogenic NOD*ShiLt* splenocytes into NOD*scid* recipients aged 6–7 weeks old. N = 13–14/recipient group (1 × 10^6^–2 × 10^7^ cells/mouse). ■ NOD + ALT into NOD*scid* recipients; ● NOD into NOD*scid* recipients.

We next examined if ALT affected systemic cellular immune responses. After 28–32 days of ALT therapy, we determined the cytokine-response following an ovalbumin (OVA) challenge in splenocytes from pre-immunised NOD*ShiLt* mice one week after an ovalbumin immunisation (given at day 21–25 of treatment). Quantification of interferon-γ (IFN-γ) producing splenocytes by ELISpot revealed no differences between untreated mice and ALT-treated mice (Figure 3A). Consistent with this, the ability to adoptively transfer diabetes to NOD*scid* recipients by splenocytes from 28–32-day ALT-treated NOD*ShiLt* donors (9/13 diabetic; Figure 3B) was similar to that of control splenocytes (10/14 diabetic). The age at transfer and slight variances in cell number did not associate with diabetes transfer (*p* = 0.06 and *p* = 1.0, respectively; cox regression). This suggests short-term ALT therapy does not compromise systemic leukocyte function.

### 2.7. Immunopeptidomics and Proteomics of Pancreatic Beta Cells Following ALT Therapy

**Figure 4:**
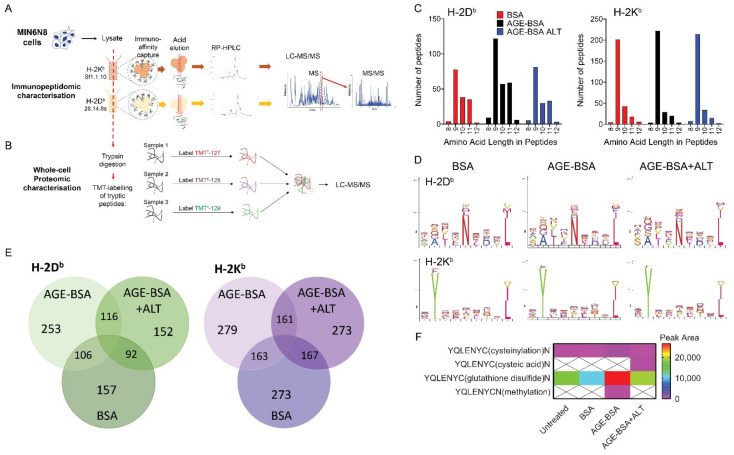
Immunopeptidomic and whole-cell proteomic characterisation of murine NOD β-cells. NOD*ShiLt* derived MIN6N8 β-cells were treated overnight with unmodified BSA (BSA; 100 µg/mL), AGE-modified BSA (AGE-BSA; 100 µg/mL), with or without alagebrium chloride (AGE-BSA+ALT; 40 µM) or untreated, with ALT vehicle control (PBS) or with BSA+ALT (Figures S7 and S8). Pooled cells (total 2 × 10^7^ cells, pooled from *n* = 4175 cm^2^ tissue culture flasks) from each treatment were lysed and antibodies against MHC Class 1a H-2K^b^ (clone sf1.1.10) and H-2D^b^ (clone 28.14.8 s) used to capture and identify presented peptides. Peptide complexes were run on RP-HPLC and identified via LC-MS/MS. Schematic of (**A**) immunopeptidomic and (**B**) whole-cell proteomic workflow used. (**C**) Number and length of MHC Class 1a H-2D^b^ (left) and H-2K^b^ (right) captured peptides isolated from BSA (red), AGE-BSA (black) and AGE-BSA+ALT (blue) treated MIN6N8 cells. (**D**) MHC Class Ia bound amino acid motifs of H-2D^b^ (top) and H-2K^b^ (bottom) peptides isolated. (**E**) Number of common H-2D^b^ (green; left) and H-2K^b^ (purple; right) immunocaptured MHC Class Ia associated peptides. (**F**) Modifications of insulin peptides precipitated with MHC Class Ia H-2D^b^ antibody from MIN6N8 cells. Scale bar shows peak area.

We have previously demonstrated in vitro that advanced glycation end product modified BSA (AGE-BSA) impairs insulin secretion and adenosine triphosphate (ATP) production in MIN6N8 cells, which was reversed by ALT treatment [11]. To examine whether chronic exposure to a high AGE environment and ALT treatment resulted in changes in the peptide repertoire presented by MHC class Ia (immunopeptidome), bound peptides (pMHC-I; H-2D^b^ and H-2K^b^) were immunoaffinity captured from treated MIN6N8 and sequenced via LC-MS/MS (Figure 4A). The complete dataset for both D^b^ and K^b^ are shown for untreated and treated MIN6N8 cells in Tables S1 and S2, respectively, and are accessible via the PRIDE data repository (see Data Availability Statement). Overall, no major changes in the immunopeptidome were observed during the various treatments with all peptides exhibiting allele specific consensus binding motifs. As expected, longer peptides were captured bound to H-2D^b^ than H-2K^b^, with the H-2D^b^ peptides tending to be of longer chain length with AGE-BSA treatment (Figure 4C), when compared with either unmodified BSA (control) or AGE-BSA+ALT. H-2K^b^ peptides were more abundant but did not appear to differ among groups. H-2D^b^ and H-2K^b^ captured peptides were predominantly of 8–12 amino acids in length (Figure 4C) with similar bound motifs (Figure 4D), regardless of treatment (Figure S7A,B). Irrespective of the MHC-I class, the majority of H-2D^b^ and H-2K^b^ peptides that presented in each treatment, were found in at least one other condition (Figure 4E*;* Figure S7C), with differences likely reflecting subtle variation between experiments rather than significant changes to the bound peptide repertoire. The 8-mer YQLENYCN peptide (InsA_14–21_) where cysteine is modified by a glutathione disulfide, was elevated during AGE-BSA treatment compared to BSA control (Figure 4F). After the addition of ALT therapy, YQLENYC(glutathione disulfide)N decreased to a level similar to that of the BSA control (Figure 4F).

**Figure 5:**
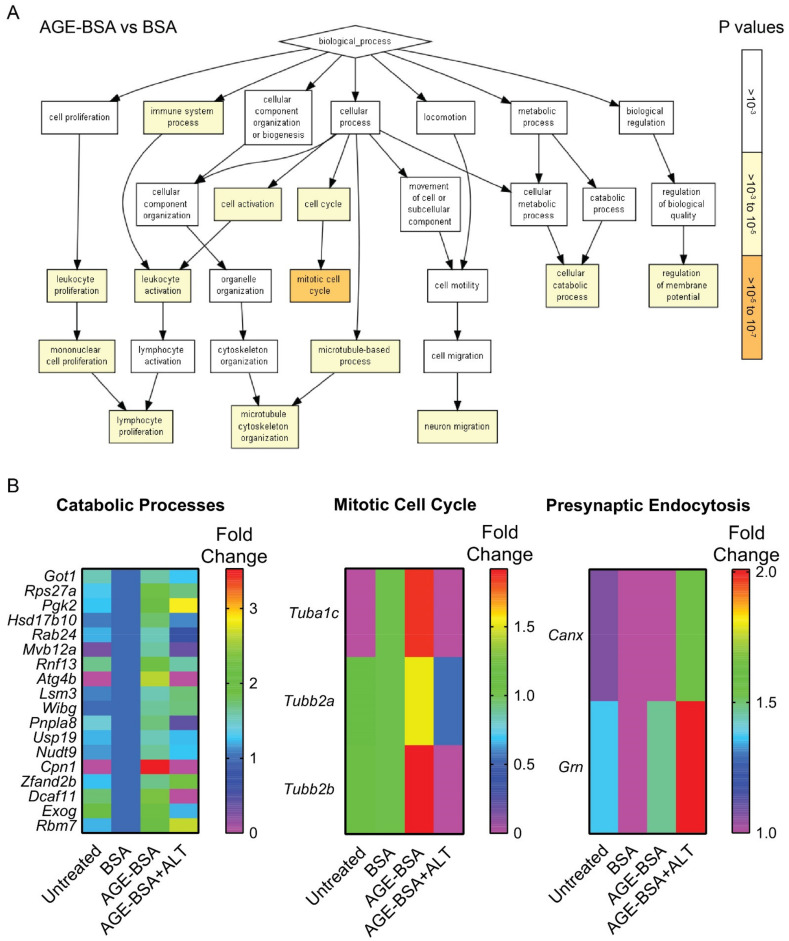
Gene Ontology enrichment analysis of biological processes in peptides isolated from MIN6N8 NOD derived beta cells. NOD*ShiLt* derived MIN6N8 beta cells were treated overnight with unmodified (BSA; 100 µg/mL), AGE-modified BSA (AGE-BSA; 100 µg/mL) without or with alagebrium chloride (AGE-BSA+ALT; 40 µM). The whole-cell proteome was performed on the cell lysate flowthrough that did not bind to MHC Class 1a antibodies. The effluents were trypsinised, peptides labelled with TMT using different isotypes and identified using LC-MS/MS. Workflow for proteomics is represented in Figure 4B. (**A**) Pathway analysis of peptides enriched following AGE-BSA vs. BSA treated MIN6N8 cells. *p* values (right; 10^−3^ to 10^−7^) shown in white-orange scale. (**B**) Heatmaps of proteins associated with three major enriched Gene Ontology pathways after AGE-BSA and AGE-BSA+ALT vs. control (BSA) MIN6N8 treated cells. Scale shows fold change.

Next, we assessed if ALT affected the proteome of the same MIN6N8 cells from which cell surface pMHC-I complexes were isolated (Figure 4B). Gene Ontology analyses of MIN6N8 β-cells cultured in a high AGE environment alone, demonstrated an enrichment in proteins associated with catabolic processes, the immune system and cell cycling compared with BSA (Figure 5A). A high AGE environment enriched for predominately Carboxypeptidase N (*Cpn1*; Figure 5B; left) and Tubulin isoforms (*Tubb*; Figure 5B; middle). With the addition of ALT, Tubulin isoforms (*Tubb*; Figure 5B; middle) and proteins involved in catabolic processes in particular Multivesicular body subunit (*Mvb12a*), Cysteine protease (*Atg4b*), Calcium-independent phospholipase (*Pnpla8*), Carboxypeptidase N (*Cpn1*) and DNA damage binding protein 1 (DDB1)- and Cullin 4 (CUL4)-associated factor 11 (*Dcaf11*) were reduced, similar to that of the BSA control (Figure 5B; left). Conversely endocytotic proteins (Figure S8) Calnexin (*Cnx*) and Progranulin (*Grn*) were enriched (Figure 5B; right) after ALT therapy.

## 3. Discussion

The present study demonstrates that short-term ALT therapy given to NOD*ShitLt* mice prior to overt disease onset and then withdrawn, does not impact systemic immune cell function or diabetes incidence, but has transient effects on β-cell function. We observed an increase in insulin secretion during glucose tolerance tests performed halfway through the ALT therapeutic period (~day 75 of life). In addition, the first phase insulin response and action during the first 15 min of the IPGTT were higher in ALT treated mice. Increases in fasted insulin as compared with control mice also persisted until one week after ALT therapy cessation. It is clear however, that these effects on β-cell function were not sufficient to arrest the development of autoimmune diabetes in ALT treated mice after withdrawal of ALT, suggesting that these β-cell effects were transient. Blood glucose concentrations were increased during oral but not intraperitoneal glucose challenges at day 75 with ALT therapy. The reasons for this remain to be ascertained in the present study. Further supporting an effect on β-cells with ALT, were experiments where pre-treatment of NOD recipients delayed diabetes development and lowered blood glucose concentrations over-time following adoptive transfer of G9C8 CD8 TCR transgenic cytotoxic lymphocytes. Circulating glucose concentrations are impacted by various pathways which are impaired in NOD mice. These include changes in peripheral insulin sensitivity [31], hypoglycaemia-glucagon feedback loops, gastric emptying, glucose intestinal absorption and the incretin effect [32]. Certainly, the higher insulin concentrations and insulin:glucose ratios suggest that peripheral insulin sensitivity may be lower in NOD mice treated with ALT as compared with control at day 75 of life. Generally AGE lowering interventions have improved insulin sensitivity in humans who are obese [33] or with type 2 diabetes [34]. However, NOD mice prediabetes often show variable insulin sensitivity which is significantly impacted by the loss of insulin secretion and so it is difficult to ascertain in the present study whether the effects of ALT on insulin, are beneficial. This may be remedied by the determination of C-peptide during OGTT which would provide specific evidence for effects on insulin secretion *per se* without being confounded by changes in insulin sensitivity. Indeed, retention of circulating C-peptide concentrations is the most common primary end-point for clinical trials in T1D treatment and prevention [35].

Gastric emptying appears to change during disease development in NOD mice and is accelerated at diabetes onset [36], however in humans, blood glucose elevations in individuals with T1D can slow gastric emptying [37,38] impacting gastrointestinal glucose absorption [39]. Glucagon-like peptide-1 (GLP-1) and gastric inhibitory polypeptide expression in the gut of NOD mice appear unchanged by hyperglycaemia [40], and although combinatory therapies with GLP-1 receptor agonists in individuals with T1D have the potential to enhance β-cell function [41], recent Phase III trials (NCT01836523, NCT02098395) have observed elevations in hypoglycaemia and hyperglycaemia with ketosis [42,43]. One explanation for a rise in glucose concentrations after the OGTT may be that production of proinsulin is contributing to overall increases in insulin at day 75 and to the end of the ALT treatment period, but limited plasma volumes throughout GTTs, prevented proinsulin from being measured. Together with the relationship between insulin secretion and lack of efficacy on progression to diabetes, our study suggests that upon removal of ALT therapy prediabetes, benefits on β-cell function are unlikely to be maintained. This therapy if given prophylactically may be best suited during the neonatal period where priming of the immune system against β-cell antigens like proinsulin [44,45] and GAD65 [45] is key in NOD mice [44] and at-risk children [45].

The reduction in insulitis observed was confirmed using flow cytometry where CD45.1^+^ cells were reduced in pancreatic digests. Here, lower proportions of CD8^+^ T cells, B cells and F4/80^+^ macrophages and higher proportions of activated F4/80^+^I-Ag7^+^ macrophages were seen. One major limitation of this work was the inability to track temporal changes to immune cells during and after ALT therapy and the inability to examine if ALT therapy impacted the expression of the cell surface receptor for AGEs, RAGE. It would be of interest to understand whether this therapy directly impacts RAGE expression on CD45.1^+^ lymphocytes locally within the pancreas. Although there is disparity as to whether ALT can mediate effects directly through RAGE, this is suggested in diabetes complications [46,47] and RAGE knockout [48,49,50] mouse models. Further, studies by our team [22] and others [51] show that inhibiting RAGE by prophylactic soluble RAGE (sRAGE) therapy, a decoy receptor that clears circulating AGEs, elevates numbers of RAGE^+^ CD4 and CD8 T cells, T_reg_ cells, DCs and macrophages in lymphoid tissue and improves Treg function [22] and prevents the transfer of diabetes by diabetogenic T cells in NOD*scid* mice [51]. With administration of ALT prediabetes, we saw no effect on IFN-γ production of OVA-stimulated splenocytes after 30 days of therapy. The lack of effect on systemic immune cell function within lymphocyte populations was further evidenced by the lack of effect of ALT-treatment of NOD donor splenocytes on diabetes incidence following adoptive transfer to NOD*scid* recipients.

Using an in vitro approach, we examined the MIN6N8 β-cell immunopeptidome; the peptide repertoire presented by MHC class Ia. The addition of ALT did not change the length of peptides recovered, which were a standard 8–12 amino acids in length as expected [52], but may have impacted their abundance, while motifs were shared amongst all treatment groups. To our knowledge, this is the first time that the immunopeptidome approach has been used to study MHC Class I peptide expression in the NOD derived MIN6N8 mouse β-cell line after AGE-lowering therapy. Although our work shows preliminary analysis and no major changes to the nature of MHC-I bound peptides, it was interesting to observe the increase of 8-mer YQLENYC(glutathione disulfide)N (InsA_14–21_) after AGE-BSA which was reduced after ALT therapy to levels similar to similar to that of BSA control. Mannering et al., previously described post-translational modifications to cysteine residues of a human insulin A-chain T cell epitope [53], and using clustered regularly interspaced short palindromic repeats (CRISPR)/Cas9 to replace murine insulin 1 with human insulin in the NOD mouse, largely protected from diabetes development [54]. The modification of glutathionylation to insulin A should be investigated further beyond this study due the abundance of glutathionylation to cysteines under oxidative stress [55].

Proteomic analysis revealed an enrichment of selected proteins after the addition of AGE-modified BSA compared to unmodified BSA. From our preliminary experiment in MIN6N8 cells, we observed upregulation in tubulin proteins which was not seen in MIN6N8 cells treated with ALT. Microtubules are critical in glucose stimulated insulin secretion [56], regulating the availability of insulin [57] and allowing the travel of insulin granules along tubulin tracks [58]. Upon microtubule destabilisation, a subset of insulin granules are released [59]. High AGE environments consistently reduce the ability of β-cells to secrete insulin which has been attributed to an increase in oxidative stress [11,13,16]. What would be interesting to substantiate in the future, is if high AGE environments result in changes or depolymerisation [14] or hyper-stabilization of the dense microtubular structure in the β-cell, resulting in insulin secretory dysfunction [59,60].

Proteomic analysis further revealed that ALT therapy enriched for calnexin and progranulin, but reduced multivesicular body subunit and cysteine protease, proteins involved in secretory [61] and autophagy [62,63,64] pathways, respectively. Calnexin is a lectin chaperone protein in the endoplasmic reticulum (ER), responsible for the folding of *N*-glycosylated proteins destined for the plasma membrane [61]. ER stress is a major contributor of β-cell fragility in autoimmune diabetes [65], and while other ER chaperone proteins are reported in the removal of mis-folded proteins [66], to our knowledge, the importance of calnexin in the β-cell has yet to be determined. Interestingly, ER stress can increase in response to progranulin administration [67] and impair insulin sensitivity in rodent models [67,68]. Within the pancreas of *MEN1* transgenic mice, progranulin was found to be a potent stimulator of β-cell proliferation in pancreatic tumours [69] and in *Grn*^−/−^mice, progranulin is vital for endolysosomal trafficking regulation [70]. Interestingly, while β-cells are high secretory cells like neurons, the role progranulins play in insulin secretion or β-cell survival is unknown.

Multivesicular body subunit forms a component of the regulatory complex that traffics ubiquinated cargos into multivesicular bodies [62,63], while cysteine protease is required for deconjugation where proteins are cleaved prior to the conjugation to phospholipids [64]. Receptor-mediated endocytosis is an important mechanism in both AGE uptake and removal [3], and in the recapture of exocytotic vesicles after insulin release in β-cells [71]. Although this is an interesting finding, conclusions on the mode of action within β-cells cannot be made. One exciting application that could further scrutinize the effect of ALT on islet cell secretion would be the rapidly developing methods that can monitor live, single vesicle fusion via two-photon microscopy [72].

Calcium-independent phospholipase hydrolyses ester bonds in phospholipids to release free fatty acids and lysophospholipids [73]. Arachidonic acid is a product generated from this reaction and can be further metabolised into proinflammatory lipid mediators. Within β-cells, calcium-independent phospholipase appears to be involved in glucose stimulated insulin secretion [74,75], proliferation and apoptosis in INS-1 cells [75] and human islets [76] and has thus been suggested as a target to improve β-cell longevity [76].

Carboxypeptidase N (*Cpn1*) is a circulating zinc metalloprotease which cleaves carboxy-terminal lysines and arginines from peptides found in the bloodstream [77]. Specific roles of carboxypeptidases have been difficult to ascertain due to the large number of protein members within the CPN family [77]. Within the islet, carboxypeptidase B1 has been shown to regulate rodent β-cell proliferation [78], carboxypeptidase E is regulated by insulin and is involved in proinsulin processing [79], carboxypeptidase H and E has been suggested as a T1D autoantibody in humans [80]. While the inhibition of carboxypeptidase M in rats reverses insulin resistance [81], and cross-talk between carboxypeptidase N and Carboxypeptidase B2 is observed during complement activation [82], the functional relevance of the downregulation of this protein with ALT is unknown.

DDB1- and CUL4-associated factors (DCAF) are proteins involved in substrate specificity for protein ubiquitination in the CUL4-DDB1 ubiquitin ligase [83]. While this ligase regulates cellular proliferation, survival and genomic integrity, DCAF11, which was downregulated by ALT therapy, has an unknown function and cellular binding partner, like most identified DCAFs [83].

Given activation of catabolic processes can be an indication of nutrient scarcity [84], the reduction of these catabolic processes by ALT therapy appear strange without the reduction of circulating blood glucose. Still, it would be interesting to understand whether ALT, which reduced subunit proteins involved in endocytic [48,49] and autophagy vesicle formation [62,63,64], has the ability to confer stress-resistance via the change in metabolic pathways associated with catabolism; fatty acid oxidation and oxidative phosphorylation [84].

The major limitation of our *omic* work is the lack of technical replicates and the ability to perform dose escalation studies. This is due to the vast number of cells required for immunopeptide precipitation and capture and the slow growth of MIN6N8 cells. Our results were from pooled replicates using previously optimised concentrations and should be interpreted with as such, however, are consistent with the functional studies that suggest a β-cell effect of ALT treatment rather than an immune mediated effect. This further emphasizes the requirement for future *omic* experiments in treated preclinical and ex vivo human islets using escalating doses of ALT to validate the effect of AGEs on the protein pathways enriched. Future studies should focus on validation of differentially expressed proteins and pathways using orthogonal validation techniques, such as multiplexed Western blotting and targeted mass spectrometry. We suggest that our database of immunopeptides and proteins (PXD025998) start as a reference point for researchers interested in therapeutic agents of advanced glycation, and its associated receptors and pancreatic islets.

Another limitation of this work is that the specific mode of action of ALT continues to remain elusive. Although first thought as a chemical cross-link breaker of α-dicarbonyl-containing compounds, evidence supporting this is lacking and the most likely mechanisms of action include metal chelation, anti-oxidant activity and bypassing the generation of MGO, previously reviewed in [85,86,87]. Certainly, we observed that circulating AGE precursors, MGO, GO and 3-DG and AGEs CML, CEL and MG-H1 were not decreased directly after cessation of ALT therapy. This may point to the therapeutic dose and exposure time to ALT, the limited number of measurements performed in mice, or simply that the reduction of intermediates or AGEs is localised to the β-cell, islet, or pancreas. AGE precursors and AGEs were not measured within islets in our study and is a clear limitation which should be prioritised in future studies [86]. The time dependency of ALT is however supported by our previous study, in which long-term prophylactic ALT therapy delayed diabetes onset [11]. It has been highlighted by this experimental work and by others [41], that one therapy is unlikely to be effective for T1D prevention. Rather, future work should focus on innovative, combinatorial approaches that could slow β-cell demise and reduce, reset and/or correct immune system function to prolong diabetes remission [41].

Taken together, these studies present a case for future work to be performed to better understand the effects of AGE lowering therapies on β-cell function and how these may be temporally determined. Certainly, the AGE-RAGE axis is gaining increasing attention as a potential pathway which can be modulated to both preserve β-cell function and arrest the development of T1D. However, our present findings suggest that prophylactic therapy, with the AGE lowering therapy, alagebrium chloride prediabetes, is not sufficiently effective in this context and other approaches should be prioritised.

## 4. Materials and Methods

### 4.1. Rodent Studies

Animal experiments were approved by an animal ethics committee (University of Queensland) and adhered to national guidelines by the National Health and Medical Research Council, Australia. Sample size calculations were estimated using a power of 0.80 and α = 0.05, where 80% of control mice were expected to develop diabetes by study end from previous studies in the animal facilities. Female NOD*ShiLt* mice were housed in the Biological Resource Facility in pathogen-free conditions at the Translational Research Institute, Brisbane Australia, and were randomly assigned to cages (*n* = 5/cage) and investigators were not blinded to treatments. Mice were given standard chow and water ad libitum, paper bedding and enrichment, and maintained on 12-h light-dark cycles, handled equally and allowed to acclimatise in the facility for at least 7 days prior to the start of procedures. Body weights and non-fasted blood glucose measurements by a glucometer (SensoCard) were taken weekly until day 100 of life, where measurements increased in frequency to at least twice weekly, when monitoring for diabetes incidence.

For diabetes incidence studies, groups of female NOD mice (*n* = 18–20/group) were either given no treatment or daily, subcutaneous injections of ALT chloride (1 mg/kg/day; Anthem Biosciences Ltd., Bangalore, Karnataka, India) for 50 days from day 50 to 100 of life. These mice were followed until diabetes was diagnosed (two consecutive, non-fasting blood glucose measurements of ≥15 m·mol/L) or until day 250 of life in the absence of diabetes. To determine the effect of ALT prediabetes, groups of female NOD mice (*n* = 10/group) were either given no treatment or daily, subcutaneous injections of ALT chloride (1 mg/kg/day; Anthem Biosciences Ltd.) for up to 40 days from day 50 of life, and were sacrificed from day 80–90 of life. To study effects on pancreatic islets, pancreatic infiltrate and splenocyte function, female NOD*ShiLt* mice were randomised (*n* = 8/group) to receive no treatment or subcutaneous injections of ALT (1 mg/kg/day; Anthem Biosciences Ltd.) for 28–32 days from day 50 of life.

Adoptive transfer studies involved either the treatment of female donor or recipient NOD*ShiLt* mice (*n* = 5–10/group) with or without subcutaneous injections of ALT (1 mg/kg/day; Anthem Biosciences Ltd.). Either treated or untreated NOD splenocytes were pooled and injected intravenously (1 × 10^6^–2 × 10^7^ cells/mouse) into 6-week old NOD.CB17-Prkdcscid/J recipient mice or NOD G9C8 cytotoxic T lymphocytes [30] were activated using recombinant insulin B15-23 peptide, and injected intravenously into 80- day-old-treated NOD*ShiLt* recipients [88]. Recipient mice were followed until diabetes diagnosis as described above or until day 250 of life in the absence of diabetes. At the end of the study or experiment end, mice were fasted and anesthetized as previously described [89].

### 4.2. MIN6N8 Cells

MIN6N8 cells (kindly provided by Professor Jun-ichi Miyazaki, Osaka University) were maintained in phenol-red free DMEM (ThermoFisher Scientific, Scoresby, VIC, Australia) containing 25 mM glucose (Sigma-Aldrich, Castle Hill, NSW, Australia), 10% *v*/*v* heat-inactivated FBS (ThermoFisher Scientific), 100 U/mL of penicillin, 100 µg/mL of streptomycin (ThermoFisher Scientific), 2 mM·L-glutamine and 71.5 µM beta mercaptoethanol (Sigma-Aldrich) [60]. Unmodified BSA (control) and AGE modified BSA were produced in-house and described elsewhere [11]. Prior to BSA (100 µg/mL), AGE-BSA (100 µg/mL) and ALT (40 µM) experimentation, MIN6N8 cells were cultured overnight in culture medium containing only 2% *v*/*v* heat-inactivated FBS.

### 4.3. Proteomic Extraction, Labelling, Detection and Quantification in MIN6N8 Cells

Refer to Supplementary Methods for full detail.

### 4.4. Metabolic and Biochemical Measurements

Glycated haemoglobin from whole blood was measured spectrophotometrically (Cobas Mira, Roche Diagnostics, Sydney, Australia) using a commercial ELISA (Crystal Chem Inc., Downers Grove, IL, USA). From fasting plasma, total insulin (Merck Millipore, Bayswater, VIC, Australia) and proinsulin (Mercodia AB, Uppsala, Sweden) were determined using commercial ELISAs following the manufacturer’s instructions. From non-fasting plasma, circulating AGEs and α-dicarbonyls were measured using an LC-MS/MS technique as described previously [33].

### 4.5. Histology and Immunohistochemistry

The tail/body of pancreata from NOD mice were fixed (10% neutral buffered formalin), dehydrated and embedded in paraffin using standard techniques. For insulitis, serial sections (*n* = 4–8/mouse, 96 µm apart) were stained using haematoxylin and eosin and quantified in a blinded fashion (26–88 islets/mouse, *n* = 4–8 mice/group), as previously described [90]. Serial sections (*n* = 2–4/mouse, 96 µm apart) were stained using anti-insulin (R&D systems, Noble Park North, VIC, Australia; clone 182410) or anti-proinsulin (R&D systems; clone 253627) as previously described [17]. Sections were imaged using an automatic slide scanner (Olympus VS120, Olympus Australia Pty Ltd., Macquarie Park, NSW, Australia). Islet area and number were quantified in a blinded fashion using Visiopharm image analysis software v4.5.6.440 (Olympus Australia Pty Ltd.). Antigen quantification from DAB^+^ areas was calculated as a reciprocal DAB intensity as detailed previously [91].

### 4.6. Flow Cytometry

Pancreata were isolated and digested to obtain single cell suspensions as previously described [92]. Antibodies against CD45.1 (A20), CD4 (GK1.5), CD8α (53-6.7), CD11b (M1/70), Cd11c (N418) and IA^g7^ (10.2.16) were purchased from Biolegend (San Diego, CA, USA). Antibodies against CD19 (ID3), CD45R (RA3-6B2) and IgM (II/41) were purchased from BD Biosciences (North Ryde, NSW, Australia). Antibodies against F4/80 (CI:A3-1) were purchased from AbD Serotec (Raleigh, NC, USA). Pancreatic cells were stained as previously detailed [92] and cytometric data were acquired in an unblinded fashion on the BD LSR II (BD Biosciences) and analysed using FlowJo software v8.

### 4.7. ELISpot Assay

Isolated splenocytes (5 × 10^5^ cells/well) were loaded into polyvinylidene difluoride ELISpot plates (Merck Millipore) which were pre-absorbed with IFN-γ antibody. Cells were stimulated with anti-CD3 (0.1 µg/mL) and maintained for 48 h in RPMI, 5% *v*/*v* FBS, 2 mM of penicillin/streptomycin/glutamine (ThermoFisher Scientific), 50 nM beta mercaptoethanol and 1 mM sodium pyruvate (ThermoFisher Scientific). ELISpot assays were developed as previously described [25] using an immune-spot plate reader (AID GmbH, Strassburg, Germany).

### 4.8. Statistical Analyses

Data was analysed for normality by either the D’Agostino-Pearson test, Shapiro-Wilk test or by viewing histograms. Gaussian distributed data are expressed as mean ± S.D and analysed using the unpaired Student’s t-test. Non-Gaussian distributed data are reported as medians with IQR and analysed by the Mann-Whitney U test, or mixed-effects model with Sidak’s post-hoc test. Correlations were analysed via linear regression and Pearson’s correlation test. Kaplan-Meier survival curves were compared by the Log-Rank test. Predicted variables associated with survival analysis were analysed via Cox regression. Frequency distributions were evaluated by chi-squared (χ^2^). Calculations were performed using graph prism (v6.05 or v8.0.1). *p* values < 0.05 were considered statistically significant.

## 5. Conclusions

Overall, our data demonstrates that short duration of ALT therapy does not extend β-cell function, inhibit experimental autoimmune diabetes or effect systemic immune function. Future studies should dissect whether co-administration of immunomodulatory therapies with ALT during the neonatal period can have an additive protective effect and what cellular processes are changed in response to ALT in islets at the single cell level using sophisticated microscopic and *omic* approaches.

## Figures and Tables

**Table 1:** Metabolic parameters in pre-diabetic NOD mice following short term AGE lowering therapy. Female NOD*ShiLt* (NOD) mice from day 50 to 100 of life were untreated or treated with the AGE lowering therapy, alagebrium chloride (ALT, 1 mg/kg/day, s.c). Intraperitoneal (IP) and oral tolerance tests were performed after a 5–6 h fast in response to a 2 g/kg D-glucose bolus at ~day 75 of life (*n* = 5–10/group). Area under the glucose (AUC_GLUCOSE_) or insulin curve (AUC_INSULIN_) measurements were calculated from the IPGTT and OGTT curves (0 min–120 min; see curves presented in Figure S1). First phase AUC_GLUCOSE_:AUC_INSULIN_ has been taken as 0–15 min of the glucose tolerance test. Data are reported as mean ± SD. † *p* = 0.002 vs. NOD (Unpaired Student’s *t*-test); ‡ *p* < 0.0001 vs. NOD (Mann-U Whitney); * *p* = 0.02 vs. NOD (Unpaired Student’s *t*-test).

	IP Glucose Tolerance Test			Oral Glucose Tolerance Test			Fasting Insulin (p·mol/L)	Fasting Proinsulin (p·mol/L)
	AUC_GLUCOSE_ (m·mol/L/min)	AUC_INSULIN_ (n·mol/L/min)	AUC_INSULIN_: AUC_GLUCOSE_	AUC_GLUCOSE_ (m·mol/L/min)	AUC_INSULIN_ (n·mol/L/min)	AUC_INSULIN_: AUC_GLUCOSE_		
NOD	923.9 ± 81.4	37.3 ± 18.0	0.02 ± 0.008	903.5 ± 58.5	10.2 ± 0.6	0.03 ± 0.008	411.9 ± 391.3	14.0 ± 10.0
NOD + ALT	868.5 ± 45.5	75.8 ± 28.7 †	0.12 ± 0.04 ‡	1036.0 ± 78.9 *	11.8 ± 1.2 *	0.03 ± 0.004	398.4 ± 240.8	16.2 ± 7.6

**Table 2:** Biochemical parameters in pre-diabetic NOD mice following short term AGE lowering therapy. Female NOD*ShiLt* (NOD) mice from day 50 to 100 were untreated or treated with the AGE lowering therapy alagebrium chloride (ALT, 1 mg/kg/day, s.c). Non-fasting insulin concentrations and glycated haemoglobin (%) at baseline (day 50), after 30 days of treatment (day 80) and 7 days after cessation of therapy (day 107) are reported as median (IQR). Time courses were analysed using a mixed-effects model with Sidak post-hoc correction for multiple comparisons. ^a^ Non-fasting blood glucose concentrations and body weight are reported as slopes from linear regression (95% CI) across the study period until the end of the study period (day 250 of life).

	Non-Fasting Insulin (ng/mL)			Glycated Haemoglobin (%)			Non-Fasting Blood Glucose ^a^	Body Weight ^a^
	Day 50	80	107	Day 50	80	107		
NOD	0.34 (0.24–0.51)	0.31 (0.26–0.45)	0.30 (0.25–0.47)	3.12 (2.5–3.7)	4.4 (2.7–5.2)	3.8 (2.9–5.2)	0.0059 (0.002–0.010)	0.0429 (0.040–0.048)
NOD + ALT	0.37 (0.27–0.57)	0.32 (0.26–0.74)	0.44 (0.25–1.3)	2.9 (2.5–3.6)	3.4 (2.5–4.1)	3.2 (3.0–4.0)	0.0101 (0.005–0.016)	0.0393 (0.036–0.042)

## Data Availability

Availability of the immunopeptidome and proteomic datasets can be found in the PRIDE data repository, accession number: PXD025998, doi:10.6019/PXD025998.

## Supplementary Materials

The following are available online at https://www.mdpi.com/article/10.3390/metabo11070426/s1, Figure S1: ALT treatment increases insulin in response to glucose loads, Figure S2: Circulating advanced glycation end products and dicarbonyl compounds (AGE precursors) do not change throughout short-term ALT therapy but associate positively with glycated haemoglobin, Figure S3: Gating strategy for flow cytometry analysis, Figure S4: T and B cell numbers and proportions do not differ after ALT therapy in pLN, Figure S5: Conventional dendritic cell and plasmacytoid dendritic cell numbers and proportions do not differ after ALT in the pLN and spleen, Figure S6: T and B cell numbers and proportions do not differ after ALT therapy in the spleen, Figure S7: Immunopeptidomic characterisation of murine NOD beta cells, Figure S8: GO Pathway Mapping of BSA vs. AGE-BSA+ALT treated MIN6N8 cells, Table S1: K^b^ identified peptides list, Table S2: D^b^ identified peptides list, Table S3: D^b^ identified peptides list; proteins discovered in proteomic discovery, Supplementary Methods: Glucose and insulin tolerance tests, Flow Cytometry (spleen, pancreatic lymph node), Purification of MHC-peptide complexes, Identification of MHC-bound peptides using LC-MS/MS, Trypsin digestion of MIN6N8 lysates for proteomic characterization, TMT-labelling of tryptic peptides; Data Availability Statement.
