# Supplementary material for: Short Duration Alagebrium Chloride Therapy Prediabetes Does Not Inhibit Progression to Autoimmune Diabetes in an Experimental Model
Source: Metabolites. 2021 Jun 28;11(7):426. doi: 10.3390/metabo11070426 (PMC8305727; doi:10.3390/metabo11070426)
Supplement: Supplementary file 1 [file metabolites-11-00426-s001.zip › metabolites-1251767-supplementary.pdf]

Supplementary Figure S1

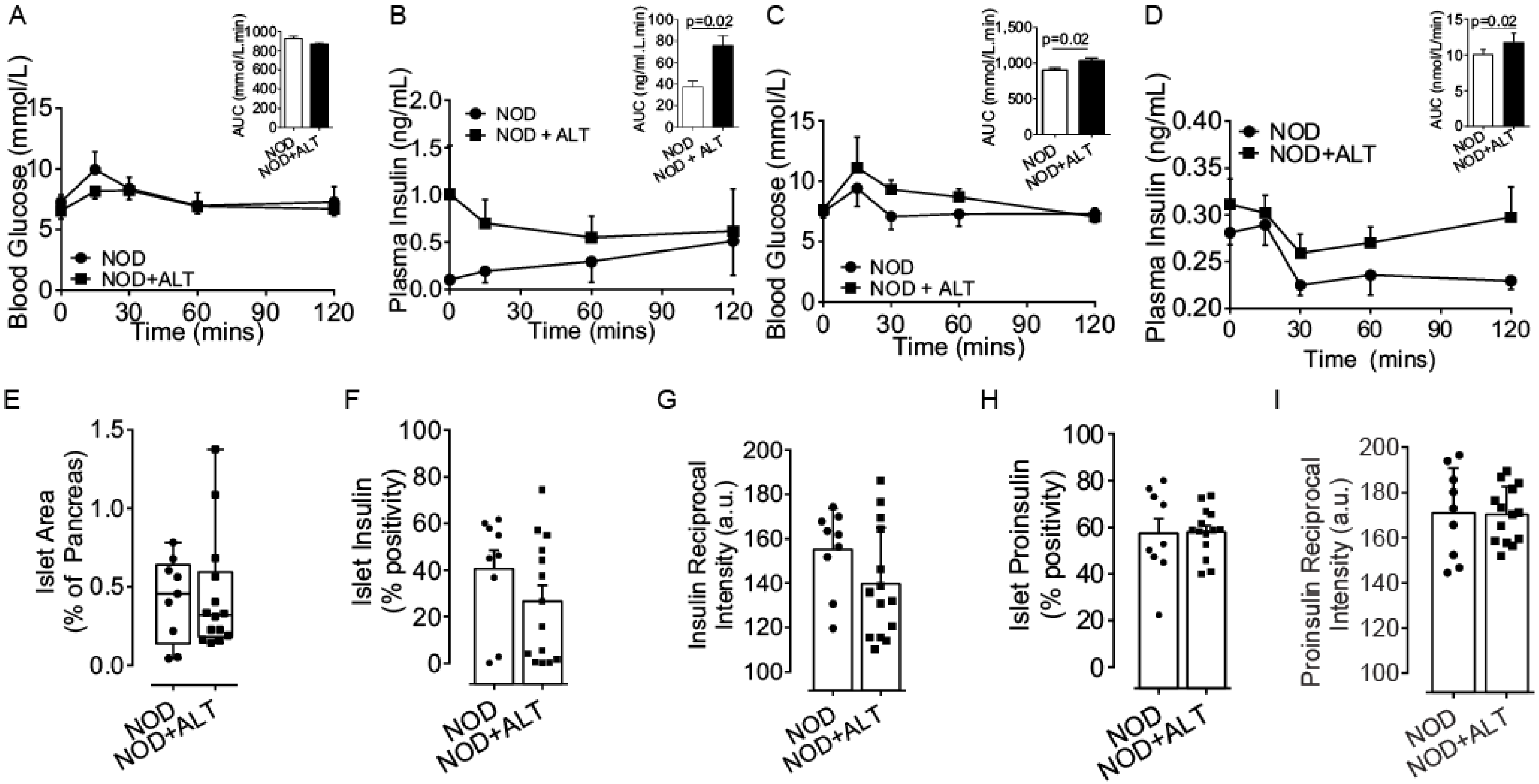

Supplementary Figure S1. ALT treatment increases insulin in response to glucose loads. NOD mice were subjected to either **A-B** IP glucose tolerance, or **C-D** oral insulin tolerance test between day 72-79 of life after alagebrium chloride treatment (n = 5-10/group) where either blood glucose **(A, C)** or plasma insulin **(B, D)** were measured. Insets: area under the glucose or insulin curves, p=0.02 vs NOD (Unpaired T-test). **(E)** Islet area, determined from proinsulin IHC staining, after 39-41 days of treatment (n = 13 - 148 islets, n = 2-4 5 µm serial sections/mouse, n = 4 mice/group). Quantification of **(F)** insulin positivity and **(G)** insulin reciprocal intensity, and **(H)** proinsulin positivity and **(I)** proinsulin reciprocal intensity in pancreatic islets after IHC staining (18 ± 15 islets/mouse, each point represents a section, n = 2-4 5 µm serials sections/mouse, n = 4 mice/group). Data is reported either as box and whisker plots reporting median, interquartile ranges and min and max values or mean ± SD.

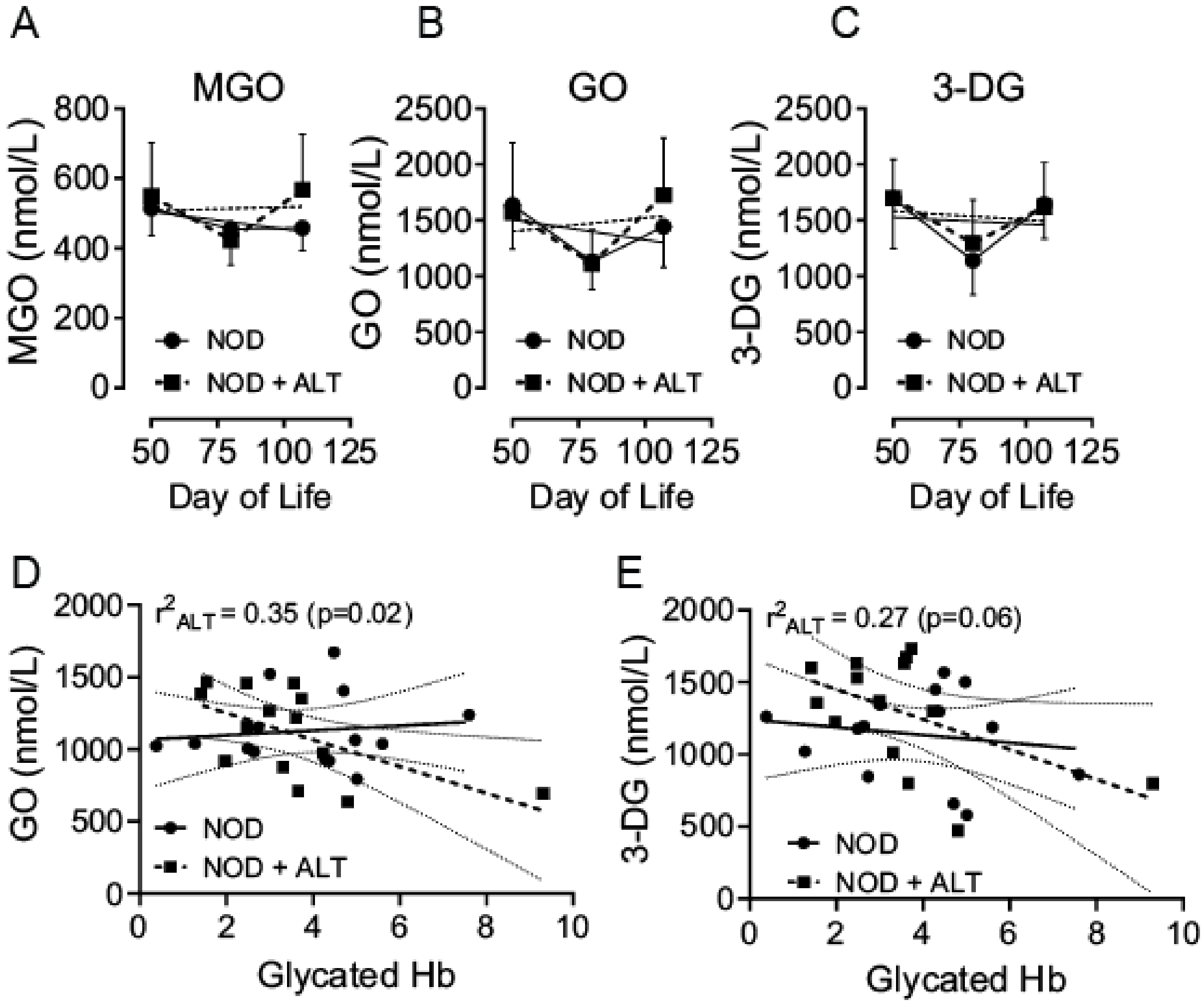

F	AGE Modifications (nmol/mmol lysine)		
	**Fasting CML**	**Fasting CEL**	**Fasting MG-H1**
NOD	110.9 ± 12.3	0.128 ± 0.03	22.8 ± 7.6
NOD + ALT	135.9 ± 46.6	0.101 ± 0.03||	18.2 ± 4.0

Supplementary Figure S2. Circulating dicarbonyl compounds (AGE precursors) and advanced glycation end products do not change throughout short-term ALT therapy but associate positively with glycated haemoglobin. Female NOD*ShiLt* (NOD) mice from day 50 to 100 of life (shaded grey) were untreated (NOD, ●, solid line) or treated with the AGE lowering therapy alagebrium chloride (ALT, 1mg/kg/day, s.c; ■, dashed line), and AGE precursors **(A)** methylglyoxal (MGO), **(B)** glyoxal (GO), and **(C)** 3-deoxyglucosone (3-DG)

were measured at 50, 80 and 107 days of life. Linear regression at day 80, after 30 days of treatment between glycated haemoglobulin (Glycated Hb) and **(D)** glyoxal (GO), and **(E)** 3-deoxyglucosone (3-DG), Linear regression (r^2) with 95% CI shown, p values of correlation analysis (Pearson test). **(F)** Circulating AGEs carboxylmethyl-lysine (CML), carboxyethyl-lysine (CEL) and methylglyoxal-derived hydroimiadazolone (MG-H1) measured at day 90, after 40 days of treatment ||p=0.056 vs NOD (Unpaired Student's T test).

Supplementary Figure S3. Gating strategy for flow cytometry analysis for (A) the pancreas (B) the pancreatic lymph node (PLN) and (C) the spleen (SPL).

(A)

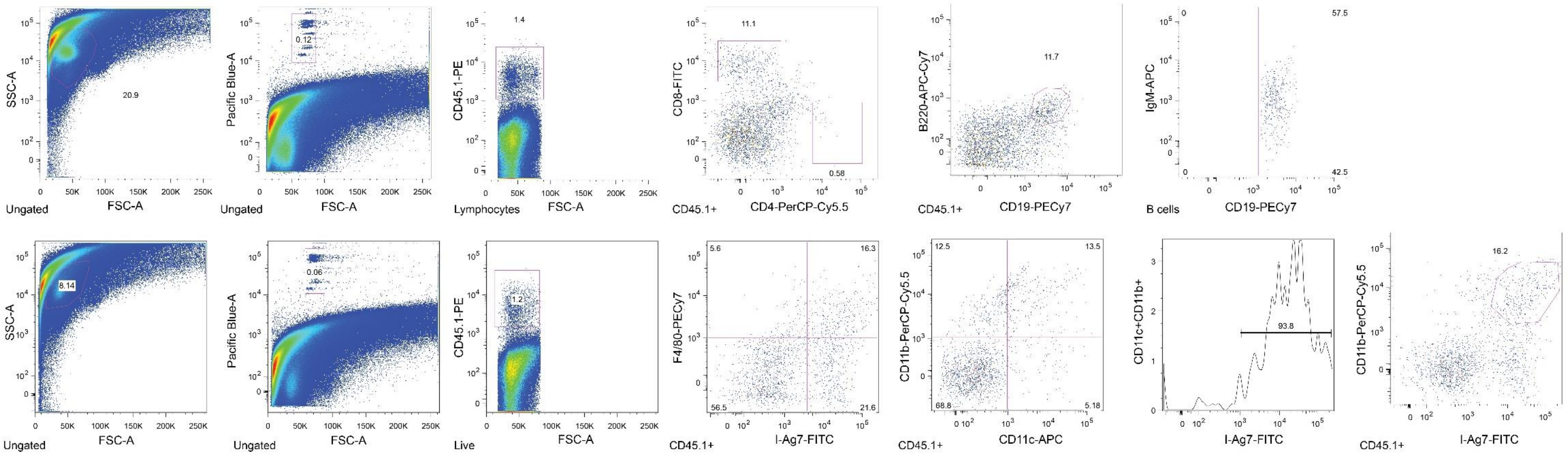

(B)

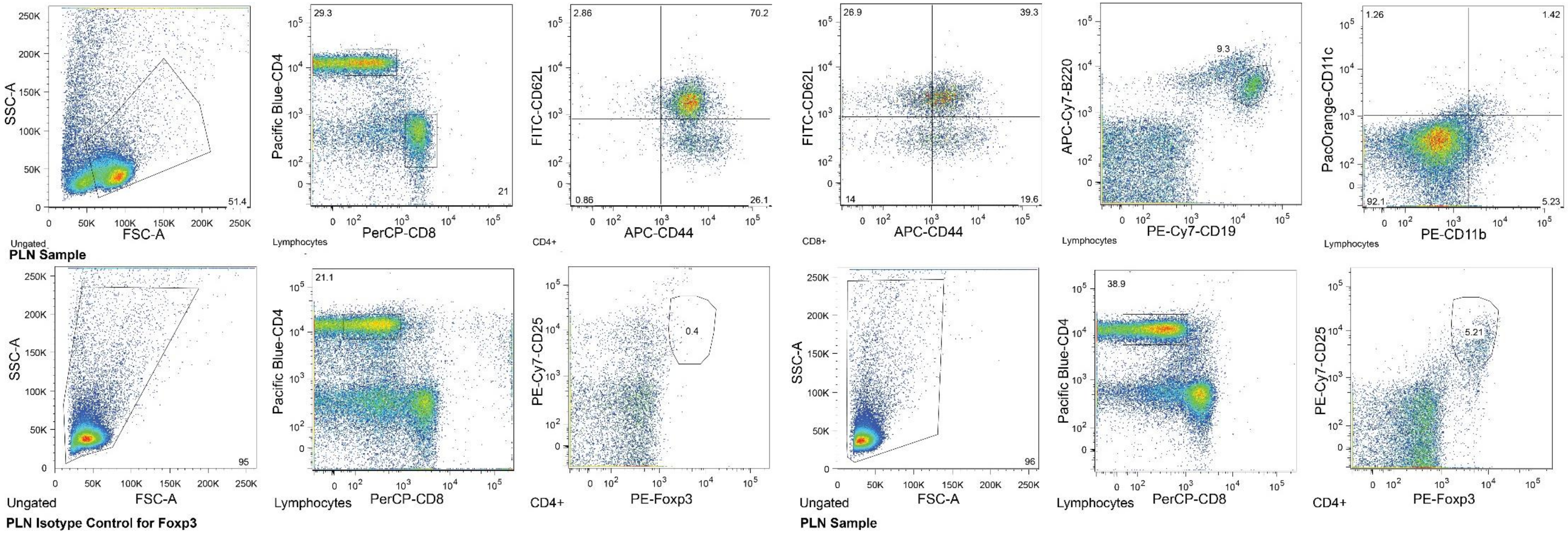

SSC-A
FSC-A
51.4
Ungated
Pacific Blue-CD4
PerCP-CD8
29.3
21
Lymphocytes
FITC-CD62L
APC-CD44
2.86
70.2
0.86
26.1
CD4+
FITC-CD62L
APC-CD44
26.9
39.3
14
19.6
CD8+
APC-Cy7-B220
PE-Cy7-CD19
9.3
Lymphocytes
PacOrange-CD11c
PE-CD11b
1.26
1.42
92.1
5.23
Lymphocytes
PLN Sample
SSC-A
FSC-A
95
Ungated
Pacific Blue-CD4
PerCP-CD8
21.1
Lymphocytes
PE-Cy7-CD25
PE-Foxp3
0.4
CD4+
PLN Isotype Control for Foxp3
SSC-A
FSC-A
96
Ungated
Pacific Blue-CD4
PerCP-CD8
38.9
Lymphocytes
PE-Cy7-CD25
PE-Foxp3
5.21
CD4+
PLN Sample

(C)

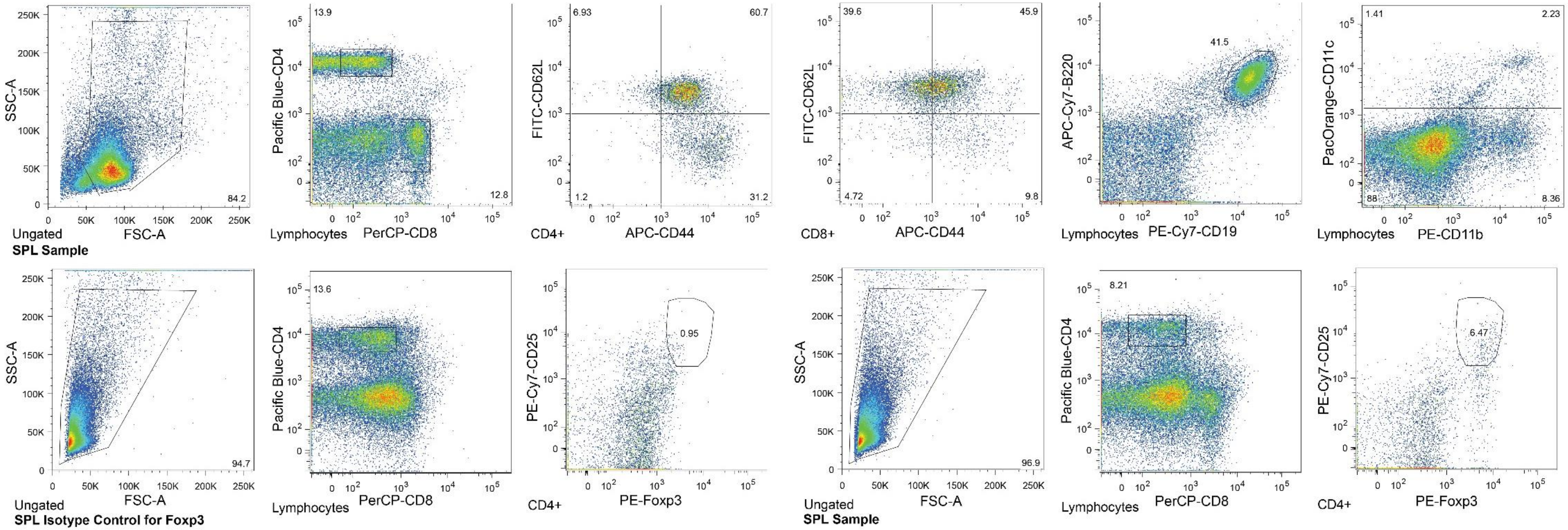
SSC-A
FSC-A
84.2
Ungated
SPL Sample
Pacific Blue-CD4
PerCP-CD8
13.9
12.8
Lymphocytes
FITC-CD62L
APC-CD44
6.93
60.7
1.2
31.2
CD4+
39.6
45.9
4.72
9.8
CD8+
APC-Cy7-B220
PE-Cy7-CD19
41.5
Lymphocytes
PacOrange-CD11c
PE-CD11b
1.41
2.23
88
8.36
Lymphocytes
94.7
SPL Isotype Control for Foxp3
13.6
PE-Cy7-CD25
PE-Foxp3
0.95
96.9
8.21
6.47

Supplementary Figure S4. T and B cell numbers and proportions do not differ after alagebrium chloride therapy (ALT) in the pancreatic lymph node (PLN).

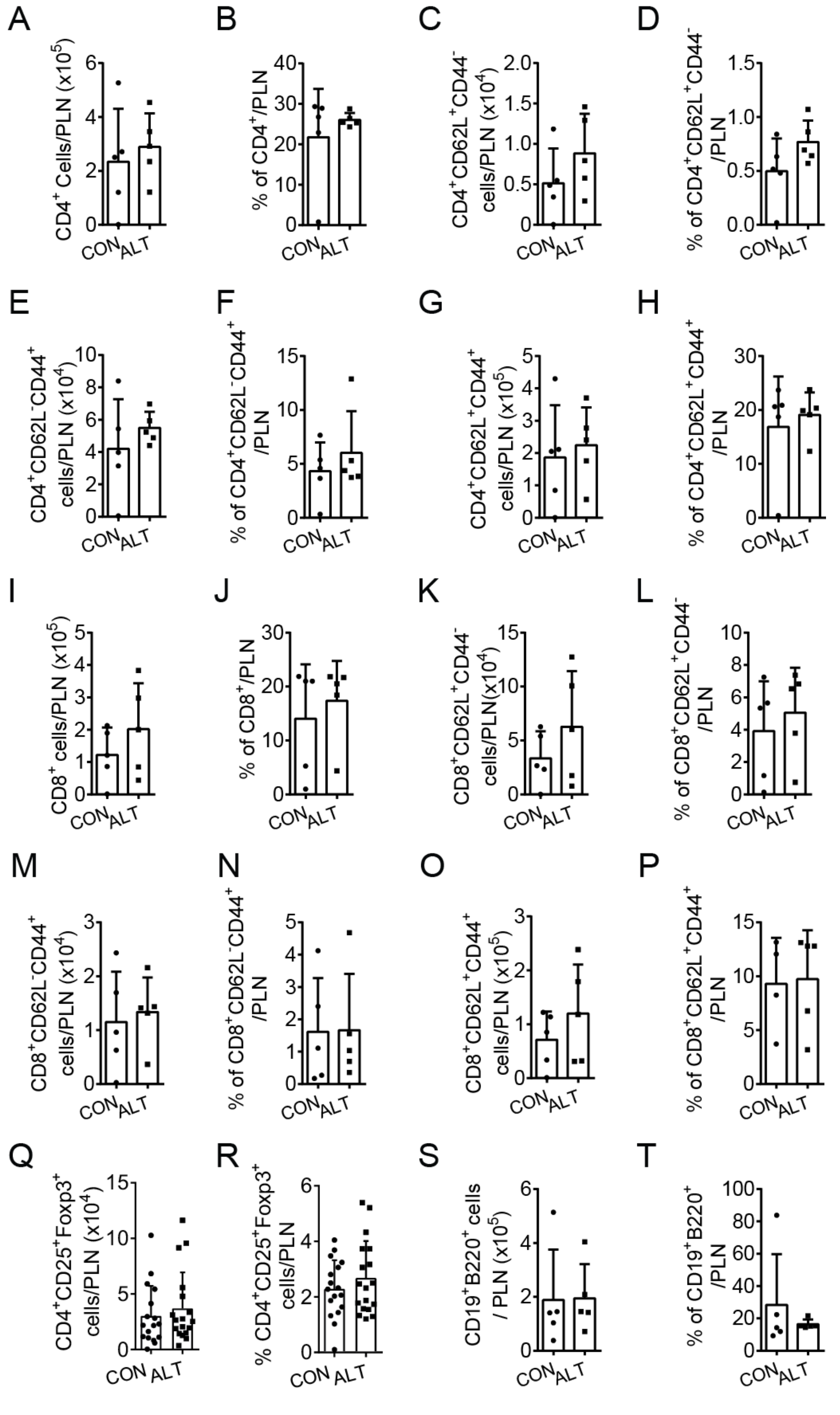

Supplementary Figure S5. Conventional dendritic cell (A, B, E, F) and plasmacytoid dendritic cell (C, D, G, H) numbers and proportions do not differ after alagebrium chloride therapy (ALT) in the pancreatic lymph node (PLN; A-D) and spleen (E-H).

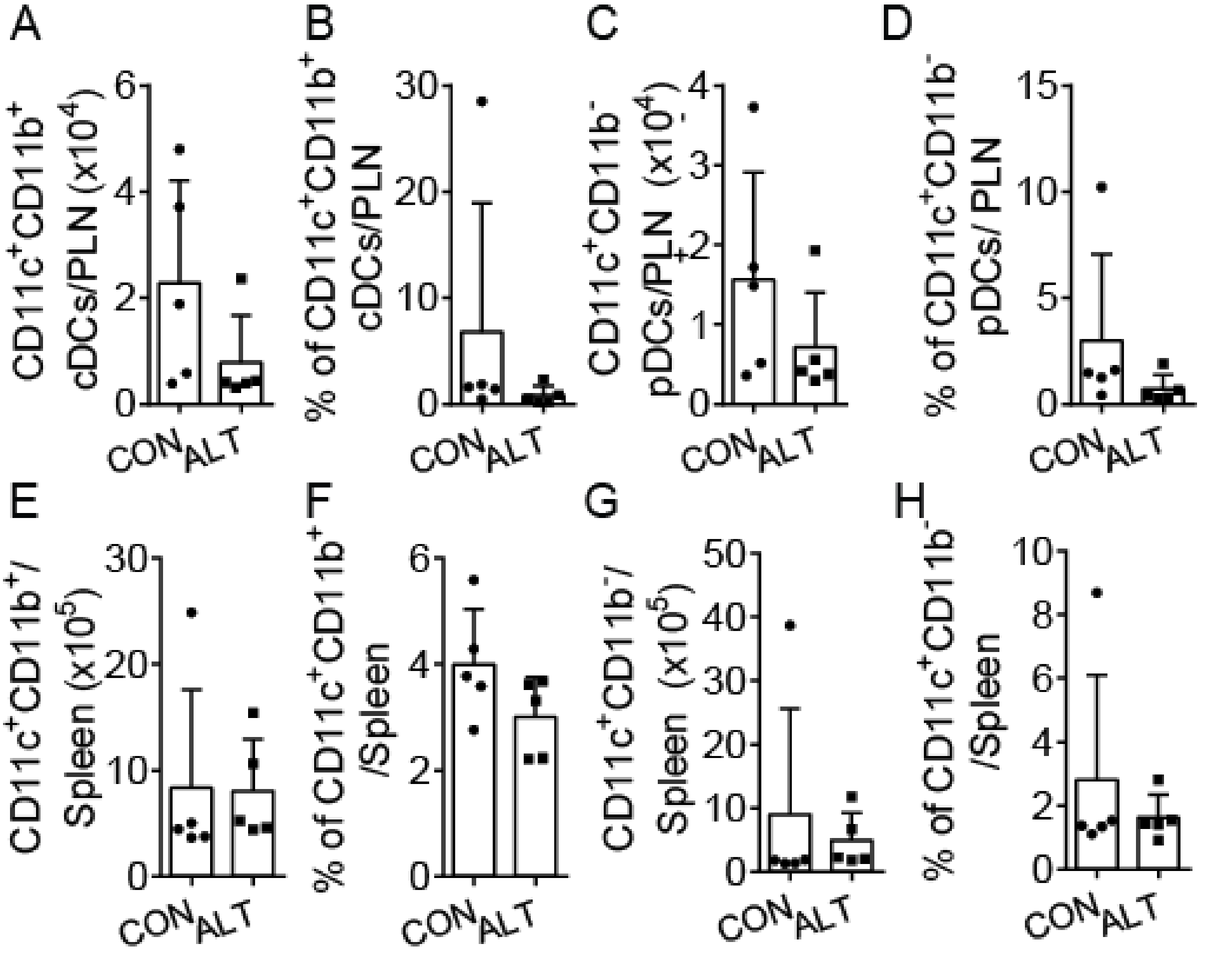

Supplementary Figure S6. T and B cell numbers and proportions do not differ after alagebrium chloride (ALT) therapy in the spleen. † p=0.02 vs NOD (Mann Whitney test)

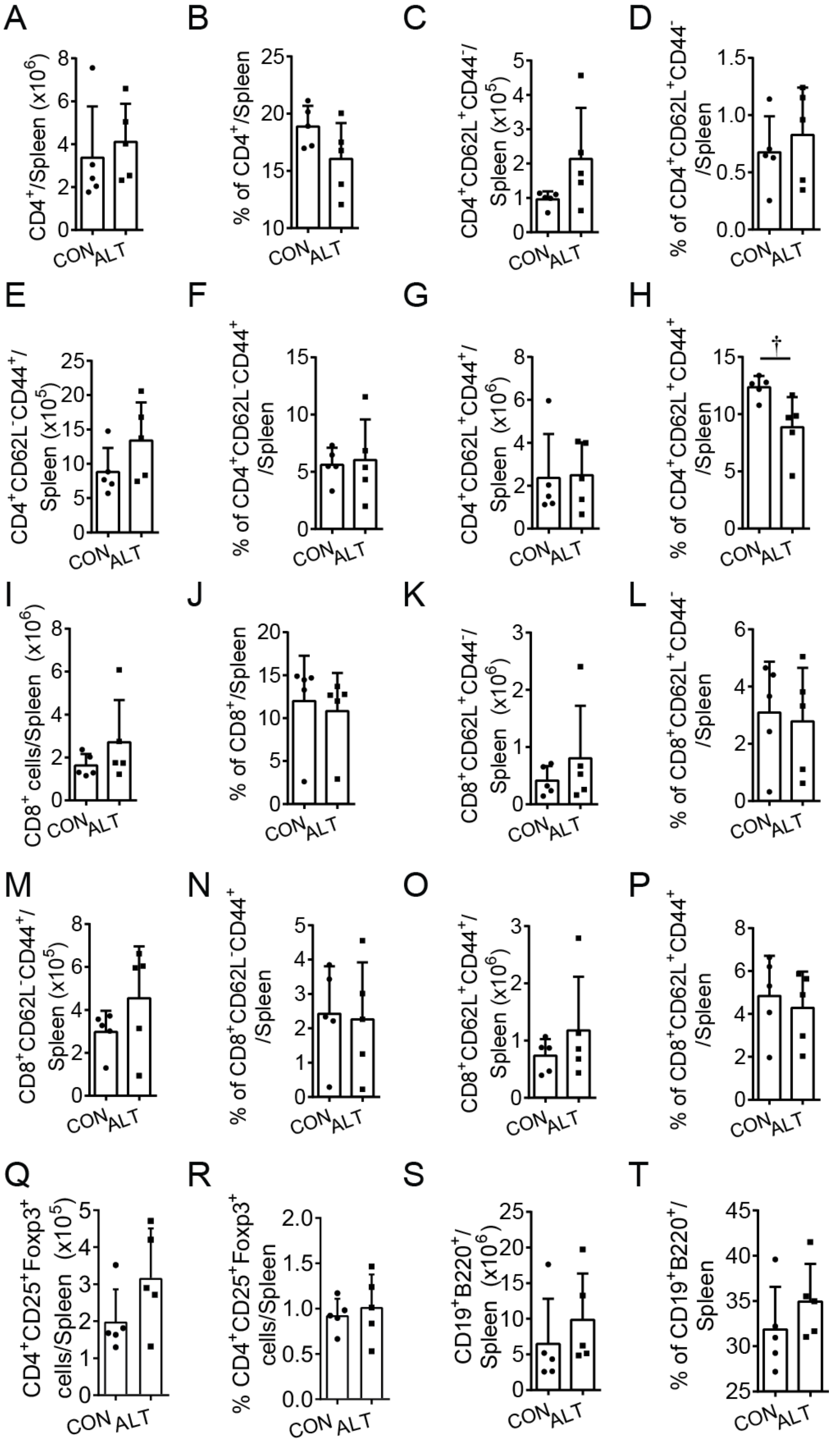

Supplementary Figure S7.

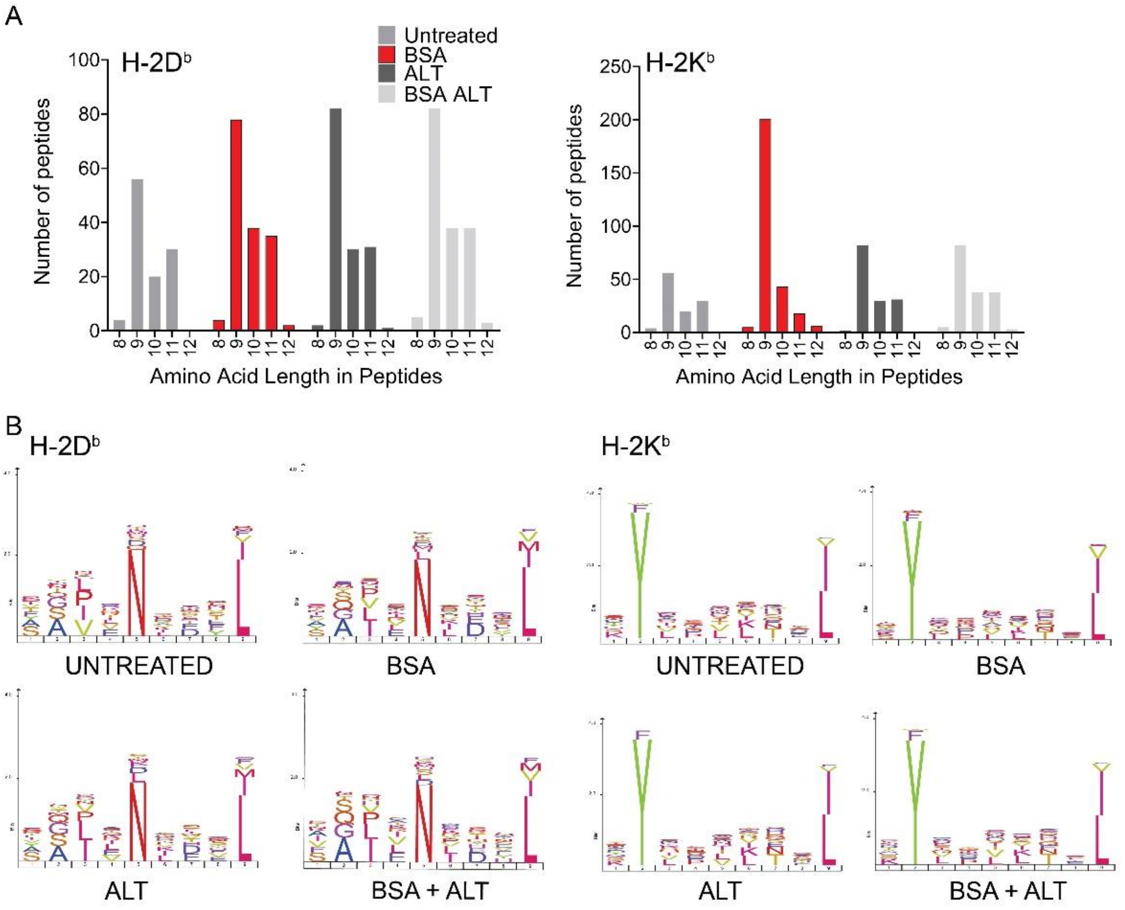

C

H-2Dᵇ

	AGE-BSA ALT (152 peptides)	AGE-BSA (253 peptides)	BSA ALT (166 peptides)	BSA (157 peptides)	ALT (146 peptides)	Untreated (110 peptides)
AGE-BSA ALT (152 peptides)						
AGE-BSA (253 peptides)	116 (40.1%)					
BSA ALT (166 peptides)	91 (40.1%)	114 (37.4%)				
BSA (157 peptides)	92 (42.4%)	106 (34.9%)	94 (41.0%)			
ALT (146 peptides)	88 (41.9%)	112 (39.0%)	95 (43.8%)	90 (42.3%)		
Untreated (110 peptides)	73 (38.6%)	82 (29.2%)	70 (34.0%)	68 (34.2%)	62 (32.0%)	

H-2Kᵇ

	AGE-BSA ALT (273 peptides)	AGE-BSA (279 peptides)	BSA ALT (193 peptides)	BSA (273 peptides)	ALT (197 peptides)	Untreated (292 peptides)
AGE-BSA ALT (273 peptides)						
AGE-BSA (279 peptides)	161 (41.2%)					
BSA ALT (193 peptides)	133 (39.9%)	123 (35.2%)				
BSA (273 peptides)	167 (44.1%)	163 (41.9%)	122 (35.5%)			
ALT (197 peptides)	142 (43.3%)	126 (36.0%)	114 (41.3%)	136 (40.7%)		
Untreated (292 peptides)	166 (41.6%)	181 (46.4%)	131 (37.0%)	171 (43.4%)	142 (40.9%)	

Supplementary Figure S7. Immunopeptidomic characterisation of murine NOD β-cells. NOD*ShiLt* derived MIN6N8 β-cells were untreated or treated with unmodified (BSA; 100 µg/mL) with or without alagebrium chloride (40 µM; BSA+ALT) or alagebrium chloride (ALT) alone. Cells were lysed and antibodies against MHC Class 1a H-2K^{b} (clone sf1.1.10)

and H-2D^b (clone 28.14.8s) used to capture and identify presented peptides. Peptide complexes were run on RP-HPLC and identified via LC-MS/MS. The whole-cell proteome was performed on the cell lysate flowthrough that did not bind to MHC Class 1a antibodies. The effluents were trypsinised, peptides labelled with TMT using different isotypes and identified using LC-MS/MS. **(A)** Number and length of MHC Class 1a H-2K^b and H-2D^b captured peptides isolated. **(B)** MHC Class Ia bound amino acid motifs of H-2D^b and H-2K^b peptides identified in isolated peptides. **(C)** Number of common H-2D^b (top) and H-2K^b (bottom) immunocaptured MHC Class Ia associated peptides.

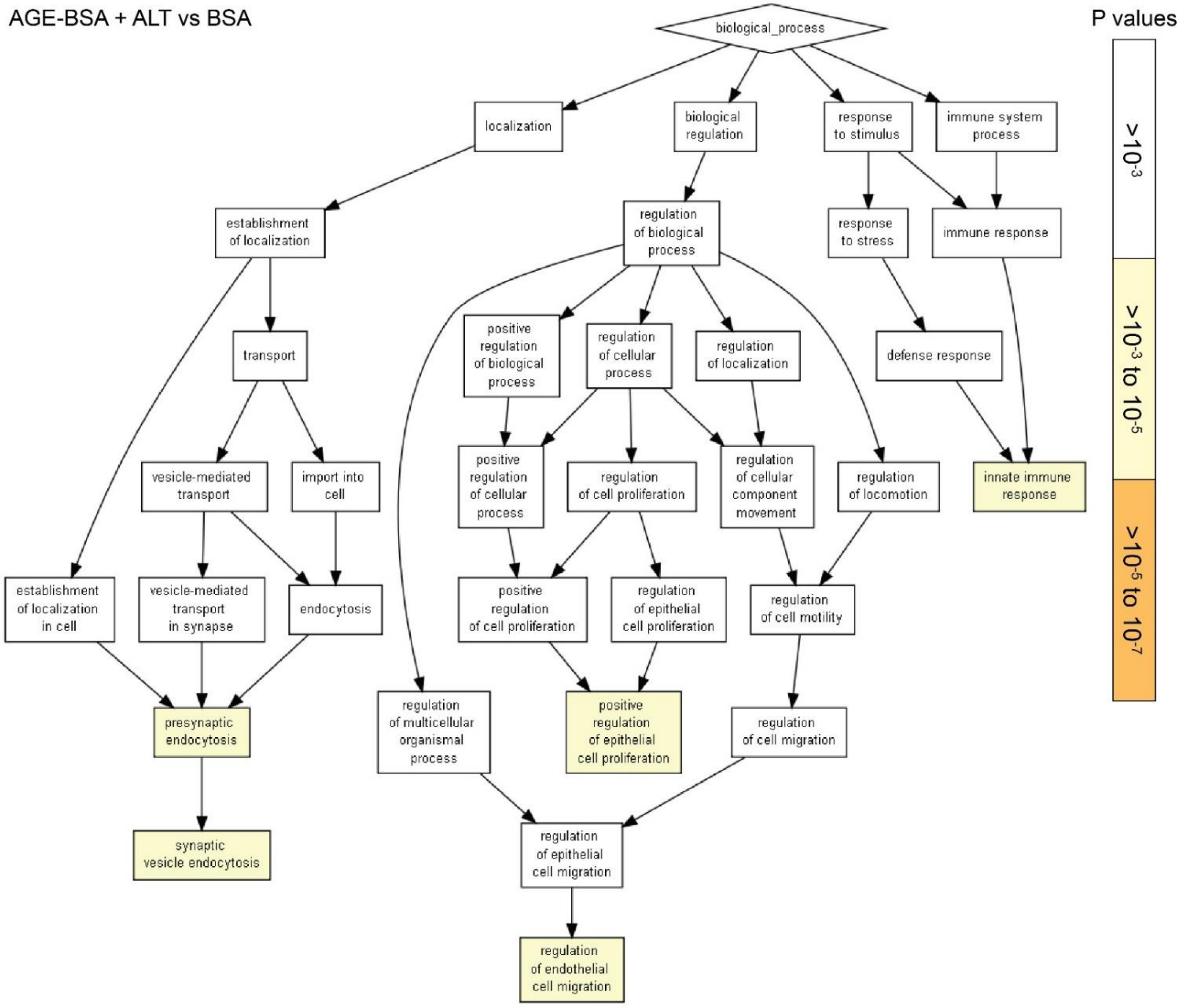

Supplementary Figure S8: GO Pathway Mapping of BSA vs AGE-BSA+ALT treated MIN6N8 cells. NOD*ShiLt* derived MIN6N8 β-cells were treated overnight with unmodified (BSA; 100 µg/mL) or modified AGE-BSA with alagebrium chloride (40 µM; AGE-BSA+ALT).

Supplementary Tables. Refer to excel supplementary files and/or PRIDE accession number PXD025998.

Supplementary Table S1. K^b identified peptides list. MHC Class I Peptide K^b-associated complexes identified in untreated (including unmodified BSA control and ALT reconstitution control), alagebrium only (ALT only), unmodified BSA control with ALT, AGE-modified BSA, and AGE-modified BSA treated with alagebrium chloride MIN6N8 cells ($2x10^7$ cells/treatment; pooled from n=4 $T175cm^2$ tissue culture flasks).

Supplementary Table S2. D^b identified peptides. MHC Class I Peptide D^b-associated complexes identified in untreated (including unmodified BSA control and ALT reconstitution control), alagebrium only (ALT only), unmodified BSA control with ALT, AGE-modified BSA, and AGE-modified BSA treated with alagebrium chloride MIN6N8 cells ($2x10^7$ cells/treatment; pooled from n=4 $T175cm^2$ tissue culture flasks).

Supplementary Table S3. Proteins discovered in proteomic discovery after trypsin digest of MIN6N8 cells following immunocapture (Figure 4B).

Supplementary Methods

Glucose tolerance tests. Tolerance tests were performed on NOD mice treated with or without ALT from day 75 – 79 of life after 4-6 hours of fasting. Glucose was given either via intraperitoneal injection (1 g of 10% w/v glucose/kg; n=10/group) or via oral gavage (2 g of 50% w/v glucose/kg; n=5/group) [1]. Blood samples and blood glucose measurements were taken prior to the glucose or insulin bolus (0 mins) and 15, 30, 60, 90 and 120 mins post-bolus using a glucometer. Plasma was isolated from whole blood and total insulin was determined using an insulin ELISA (Merck Millipore, Bayswater, Australia).

Flow Cytometry (spleen, pancreatic lymph node). Antibodies against CD4 (RM4-5), CD8α (53-6.7), CD62L (MEL-14), CD44 (IM7), CD45R (RA3-6B2), CD19 (ID3), CD11b (M1/70), CD11c (HL3), CD25 (PC61) were purchased from BD Biosciences (North Ryde, Australia). FoxP3 staining kit was used for intracellular staining and was purchased from eBiosciences (ThermoFischer Scientific, North Ryde, Australia). Spleens and pancreatic lymph nodes were passed through a 40 μm filter and washed with HBSS/2% foetal bovine serum (FBS) and erythrocytes (for spleen cells) were lysed in ammonium chloride buffer. Cells were washed in PBS/2% FBS and centrifuged (370g, 5 mins, 4°C). Cells were resuspended, counted and $1x10^6$ cells were stained with the aforementioned antibodies. Intracellular staining was performed according to the manufacturer's protocol. Cells were washed in PBS/2% FBS and centrifuged. Cells were fixed using BD Cytofix (BD Biosciences) according to the manufacturer's instructions. Cytometric data were acquired in an unblinded fashion on a LSRII flow cytometer (BD Biosciences). Absolute cell numbers were estimated using total cell counts from each organ.

Purification of MHC-peptide complexes. Treated and untreated MIN6N8 cell pellets were respectively lysed in 0.5% IGEPAL, 50 mM Tris-HCl pH 8.0, 150 mM NaCl and protease

inhibitors (Complete Protease Inhibitor Cocktail Tablet; Roche Molecular Biochemicals) at a density of 2 x 10^7 cells/mL and incubated at 4 °C for 1 hr. Lysates were cleared by ultracentrifugation (180,000 x g) and MHC-peptide complexes from each sample were respectively immunoaffinity purified in tandem using solid-phase-bound sf1.1.10 (H-$2K^b$ -specific) and 28.14.8s (H-$2D^b$ -specific) monoclonal antibodies as previously described [2]. Bound complexes were eluted by acidification with 10% acetic acid. The mixture of peptides, MHC heavy chains and ß2-microglobulin was fractionated on a 4.6-mm internal diameter X 50-mm long monolithic C_{18} reverse-phase high-performance liquid chromatography column (Chromolith SpeedROD; Merck) using an ÄKTAmicro HPLC system (GE Healthcare), with a mobile phase consisting of buffer A (0.1 % trifluoroacetic acid) and buffer B (80% acetonitrile, 0.1 % trifluoroacetic acid). Lysates devoid of pMHC complexes were each kept for further whole-cell proteomic characterisation.

Identification of MHC-bound peptides using LC-MS/MS. Peptide-containing fractions were vacuum concentrated and loaded onto a microfluidic trap column packed with ChromXP C_{18}-CL 3-µm particles (300 Å nominal pore size; equilibrated in 0.1% formic acid/5% acetonitrile) at 5 µL/min using an Eksigent NanoUltra cHiPLC system. An analytical (15 cm x 75 µm ChromXP C_{18}-CL 3) microfluidic column was then switched in line and peptides separated by linear gradient elution of 0-80% acetonitrile over 90 min at a flowrate of 300 nL/min. Separated peptides were analysed with an AB SCIEX 5600^+ TripleTOF mass spectrometer equipped with a Nanospray III ion source and accumulating up to 30 MS/MS spectra per second. MS/MS spectra were analysed via PEAKS studio X software. MS files were imported into PEAKS X with the following settings: parent mass error tolerance was set at 50 parts per million with a fragmentation mass error of 0.1 Da. An initial de novo search of all MS/MS spectra against peptide sequences was performed followed by a specific search against the human proteome database with oxidation of methionine selected as a variable PTM

followed by PEAKS PTM analysis. A false discovery rate (FDR) of 5% was implemented [3] and all resulting peptides were exported. Sequence motifs were identified using the IceLogo server (https://iomics.ugent.be/icelogoserver/)[4].

Trypsin digestion of MIN6N8 lysates for proteomic characterisation. Treated or untreated MIN6N8 lysate that has been devoid of pHLA complexes following immunoaffinity capture was subjected to trypsin digestion. To recover proteins from detergent in the lysate, proteins were precipitated by incubation with ice-cold acetone for 1 hr. Precipitated proteins were pelleted by centrifugation and reconstituted in 50 mM NH_4HCO_3, pH checked to be between pH 7-8. Resolubilized proteins were then treated with the reducing agent tris(2-carboxyethyl)phosphine (TCEP) to a final concentration of 1 mM and heated at 60 ℃ for 30 min. To alkylatate cysteine residues, iodoacetamide were added to a final concentration of 25 mM and incubated in the dark for 30 min. Trypsin (Sigma-Aldrich) was added into protein mixture at a trypsin:protein ratio of 1:100 and incubated overnight at 37 ℃. Tryptic peptides were fractionated on a a 4.6-mm internal diameter X 50-mm long monolithic C_{18} reverse-phase high-performance liquid chromatography column (Chromolith SpeedROD; Merck) using an ÄKTAmicro HPLC system (GE Healthcare), with a mobile phase consisting of buffer A (0.1 % trifluoroacetic acid) and buffer B (80% acetonitrile, 0.1 % trifluoroacetic acid). Fractionated tryptic peptides for each treated group were pooled into 1 final sample, vacuumed concentrated and peptides subjected to TMT-labelling.

TMT-labelling of tryptic peptides. The labelling reaction was conducted using multiplex TMT reagents (Thermo Scientific). For multiplex comparative and quantitative proteomics 100 µg of each cell lysate was digested with trypsin and labelled with Tandem Mass Tag (TMT) reagents according to the manufacturer's protocol (Thermo Fisher Scientific). After labelling, samples were combined in equal amounts and run on the Orbitrap Fusion™ Tribrid™ mass spectrometer (Thermo Fisher Scientific) for peptide sequence analysis. MS/MS spectra were

analysed via PEAKS studio X software. MS files were imported into PEAKS X with the following settings: parent mass error tolerance was set at 50 parts per million with a fragmentation mass error of 0.1 Da. An initial de novo search of all MS/MS spectra against peptide sequences was performed followed by a specific search against the human proteome database with Carbamidomethylation of Cysteine and TMT of N-terminal selected as a fixed PTM followed by PEAKS Quant analysis. A false discovery rate (FDR) of 1% was implemented and all resulting peptides were exported. Gene Ontology was analysed using **G**ene **O**ntology en**RI**chment ana**L**ysis and visua**L**iz**A**tion tool (Gorilla; http://cbl-gorilla.cs.technion.ac.il/) [5, 6].

Data Availability Statement. The datasets presented in this study can be found in online repositories. The names of the repository/repositories and accession number can be found below: PRIDE repository, accession number: PXD025998, doi: 10.6019/PXD025998.

References

1. Andrikopoulos, S., et al., *Evaluating the glucose tolerance test in mice.* American Journal of Physiology - Endocrinology And Metabolism, 2008. 295(6): p. E1323-E1332.
2. Purcell, A.W., S.H. Ramarathinam, and N. Ternette, *Mass spectrometry–based identification of MHC-bound peptides for immunopeptidomics.* Nature Protocols, 2019. 14(6): p. 1687-1707.
3. Faridi, P., et al., *A subset of HLA-I peptides are not genomically templated: Evidence for cis- and trans-spliced peptide ligands*. Sci Immunol. 2018 Oct 12;3(28):eaar3947.
4. Colaert, N., et al., *Improved visualization of protein consensus sequences by iceLogo.* Nature Methods, 2009. 6(11): p. 786-787.
5. Eden, E., et al., *Gorilla: A Tool For Discovery And Visulization of Enriched GO Terms in Ranked Gene Lists.* BMC Bioinformatics, 2009. 10:48.
6. Eden, E., et al., *Discovering Motifs in Ranked Lists of DNA Sequences*. PLoS Computational Biology, 2007. 3(3)339.
